# Response of *Fusarium pseudograminearum* to Biocontrol Agent *Bacillus velezensis* YB-185 by Phenotypic and Transcriptome Analysis

**DOI:** 10.3390/jof8080763

**Published:** 2022-07-22

**Authors:** Jie Zhang, Wenqian Zhu, Paul H. Goodwin, Qitong Lin, Mingcong Xia, Wen Xu, Runhong Sun, Juan Liang, Chao Wu, Honglian Li, Qi Wang, Lirong Yang

**Affiliations:** 1 Institute of Plant Protection Research, Henan Academy of Agricultural Sciences, Henan Biopesticide Engineering Research Center, Henan Agricultural Microbiology Innovation Center, Zhengzhou 450002, China; zhangjie@hnagri.org.cn (J.Z.); zhuwenqian@hnagri.org.cn (W.Z.); linqitong@hnagri.org.cn (Q.L.); xiamingcong@hnagri.org.cn (M.X.); xuwen@hnagri.org.cn (W.X.); sunrunhong@hnagri.org.cn (R.S.); liangjuan@hnagri.org.cn (J.L.); wuchao@hnagri.org.cn (C.W.); 2 School of Environmental Sciences, University of Guelph, Guelph, ON N1G 2W1, Canada; pgoodwin@uoguelph.ca; 3 College of Plant Protection, Henan Agricultural University, Zhengzhou 450002, China; honglianli@henau.edu.cn; 4 Department of Plant Pathology, College of Plant Protection, China Agricultural University, Beijing 100193, China; wangqi@cau.edu.cn

**Keywords:** Fusarium crown rot, *Bacillus velezensis*, biocontrol, RNA-seq

## Abstract

The use of biological control agents (BCAs) is a promising alternative control measure for Fusarium crown rot (FCR) of wheat caused by *Fusarium pseudograminearum*. A bacterial strain, YB-185, was isolated from the soil of wheat plants with FCR and identified as *Bacillus velezensis*. YB-185 exhibited strong inhibition of *F. pseudograminearum* mycelial growth and conidial germination in culture. Seed treatment with YB-185 in greenhouse and field resulted in reductions in disease by 66.1% and 57.6%, respectively, along with increased grain yield. Microscopy of infected root tissues confirmed that YB-185 reduced root invasion by *F. pseudograminearum*. RNA-seq of *F. pseudograminearum* during co-cultivation with *B. velezensis* YB-185 revealed 5086 differentially expressed genes (DEGs) compared to the control. Down-regulated DEGs included genes for glucan synthesis, fatty acid synthesis, mechanosensitive ion channels, superoxide dismutase, peroxiredoxin, thioredoxin, and plant-cell-wall-degrading enzymes, whereas up-regulated DEGs included genes for chitin synthesis, ergosterol synthesis, glutathione S-transferase, catalase, and ABC transporters. In addition, fungal cell apoptosis increased significantly, as indicated by TUNEL staining, and the scavenging rate of 2,2′-Azino-bis(3-ethylbenzothiazoline-6-sulfonic acid) diammonium salt radical cation (ABTS·+) in the fungus significantly decreased. Thus, *F. pseudograminearum* may be trying to maintain normal cell functions by increasing cell wall and membrane synthesis, antioxidant and anti-stress responses, detoxification of bacterial antimicrobial compounds, and transportation of damaging compounds from its cells. However, cell death and free radical accumulation still occurred, indicating that the responses were insufficient to prevent cell damage. *Bacillus velezensis* YB-185 is a promising BCA against FCR that acts by directly damaging *F. pseudograminearum,* thus reducing its ability to colonize roots and produce symptoms.

## 1. Introduction

Wheat (*Triticum aestivum* L.) is a major food crop, susceptible to a variety of diseases [1]. Fusarium crown rot (FCR) is one of the most destructive soil-borne wheat diseases in many arid and semi-arid cropping regions of the world [2]. Symptoms are typically a crown rot at the base of the stem and a white head, leading to significant grain loss [3]. It can also result in mycotoxin contamination [4]. In recent years, FCR has become more prevalent, partly due to more infected debris with the use of minimum tillage [2]. The most common pathogens of FCR are *F. pseudograminearum*, *F. culmorum,* and *F. graminearum* [5]. In China, *F. pseudograminearum* was first reported as the causal agent of FCR in Henan Province in 2012 [6], and it has become the dominant species in the winter wheat area of the Huanghuai Plain of China [7].

Despite the success of chemical fungicides in mitigating FCR damage and raising yield, more environmentally friendly alternatives are needed to address concerns about fungicides on human health and the loss of control due to the emergence of fungicide resistance [8,9]. Biological control is a promising alternative [10]. Among biological control agents (BCAs) of plant diseases, many antagonistic soil microbes have been found with antifungal activities. Examples of bacterial BCAs to control FCR of wheat include *Pseudomonas cepacia* A3R [11] and *Bacillus subtilis* YB-15 [12], both applied as a soil drench to roots, and *Bacillus mycoides* BmJ, sprayed foliarly to induce systemic acquired resistance [13]. However, there is a need for additional BCAs for FCR caused by *F. pseudograminearum*. 

In this study, bacterial strain YB-185 was isolated from soil in a wheat field heavily infested with *F. pseudograminearum* in Jiaozuo of Henan Province, China [6]. It showed considerable antimicrobial activity against *F. pseudograminearum*, reducing its ability to colonize wheat roots and reduce yield. To better understand its mode of action, RNA-seq was done on the fungus when co-cultivated with the BCA. The result is the identification and partial characterization of a new BCA strain showing considerable effectiveness in controlling FCR by inhibiting and killing cells of *F. pseudograminearum*. 

## 2. Materials and Methods

### 2.1. Strain YB-185 Isolation and Identification

Two rhizosphere soil samples were collected from wheat plants showing FCR during the milky ripe growth stage in Jiaozuo of Henan Province, China. A total of 10 g of soil was suspended in 90 mL of sterilized water in an Erlenmeyer flask. After being shaken for 10 min, the soil suspensions were then diluted in sterile water at dilutions of 10^−1^, 10^−2^, 10^−3^, 10^−4^, 10^−5^, 10^−6^, and 10^−7^. Then, 100 μL of dilutions at 10^−5^, 10^−6^, and 10^−7^ were plated onto nutrient agar (NA, peptone: 10 g, beef extract: 3 g, NaCl: 5 g, agar: 20 g, H_2_O: 1000 mL, pH 7.0–7.2) in triplicate and incubated at 30 °C for 24 h. Single colonies were transferred and then characterized by color and shape. The purified bacteria were screened for growth inhibition of *F. pseudograminearum* by the dual-culture method [14]. The *F. pseudograminearum* WZ-8A strain, isolated from infected wheat roots in Jiaozuo, was used in this study, which was shown to be highly virulent to wheat in greenhouse [7]. Briefly, plugs of *F. pseudograminearum* isolate WZ-8A were placed in the center of PDA plates, and bacterial colonies were inoculated, with 3 cm on each side of the fungal plug. After incubation at 25 °C for 7 d, the inhibition rates of all bacterial isolates against the mycelia growth of *F. pseudograminearum* were calculated [10]. One strain, coded as YB-185, which showed the strongest antifungal effect, was selected for further study. The morphology of YB-185 was determined by scanning electron microscopy (SEM) using a Hitachi SU8100 SEM microscope (Hitachi, Tokyo, Japan). Gram staining was performed [15]. The Biolog GEN III MicroPlate (Biolog, Hayward, CA, USA) was used to identify YB-185 with 94 physiological parameters, including 71 for carbon source utilization and 23 for chemical sensitivity. For 16S sequencing, genomic DNA was extracted from YB-185 using the Mini-BEST Bacterial Genomic DNA Extraction Kit Ver. 3.0 (Takara, Beijing, China). Amplification of an approximately 1.5 kb sequence of 16S rRNA was performed as per Marchesi et al. [16], and a 1.0 kb sequence of *gyrA* gene was performed as per Chun and Bae [17]. The PCR products were purified and sequenced by Sangon Biotech Co. (Shanghai, China). The sequences were deposited at GenBank (accession numbers OL305850 and OL352704). To construct phylogenetic trees, the *gyrA* and 16S rRNA sequences of seven bacterial species were downloaded from Ensembl Bacteria Home (http://bacteria.ensembl.org/index.html, accessed on 12 March 2022) and aligned with MAFFT (https://mafft.cbrc.jp/alignment/server/, accessed on 12 March 2022). Perl was used to concatenate the aligned conservative regions of the two genes, and RAxML analysis was run (https://github.com/stamatak/standard-RAxML, accessed on 12 March 2022) to construct a maximum likelihood phylogenetic tree for the combined datasets through the CIPRES web portal (http://www.phylo.org, accessed on 12 March 2022), with 1000 bootstrap iterations.

### 2.2. Antagonistic Activity of Strain YB-185 against F. pseudograminearum 

For the conidial germination assay, a 7 mm plug from a 5-day-old *F. pseudograminearum* colony was inoculated into 50 mL carboxymethyl cellulose (CMC) broth [18] and cultured at 25 °C with shaking at 150 rpm. After 5 days, conidia were harvested by filtering through a layer of Miracloth (Merck Millipore, Billerica, MA, USA) and adjusted to 10^7^ conidia/mL using a hemocytometer. Strain YB-185 was grown in NB at 30 °C shaking at 180 rpm. After 10 h, the cells were collected by centrifugation at 8000 rpm for 5 min and resuspended in sterile water. The number of colony forming units (CFU) of YB-185 was determined by dilution plating and was adjusted to 10^8^ CFU/mL with sterile water. A 1 mL conidial suspension was transferred into 20 mL PDB containing 1 mL bacterial suspension. PDB containing 1 mL conidial suspension with only sterile water added was used as control. The dual cultures were grown at 25 °C in the dark for 12 h, and the percentage of conidial germination was measured by examining 100 conidia with a microscope (×40 magnification). A conidium was considered to have germinated if the germ tube exceeded one-half the length of the conidium.

To assay the effect of YB-185 culture filtrate on mycelial growth of *F. pseudograminearum*, YB-185 was grown in 50 mL NB for 48 h at 30 °C, shaking at 180 rpm. The culture was centrifuged at 10,000 rpm for 10 min, and the supernatant passed through a 0.22 um filter. The filtrate was added to PDA at 1:2, 1:5, and 1:10 (*v*/*v*). Then, a 5 mm *F. pseudograminearum* plug was inoculated on the medium and incubated at 25 °C in the dark, with a plug grown on PDA alone as control. At 5 days, colony diameters were measured, and the inhibition percent of mycelial growth was calculated [19]. The morphology of *F. pseudograminearum* grown on PDA with 1:5 YB-185 filtrate was observed by SEM and transmission electron microscopy (TEM). For SEM and TEM, mycelia from the margin of an *F. pseudograminearum* colony on PDA containing 1:5 YB-185 filtrate were fixed in 2% glutaraldehyde for 4 h at 4 °C and rinsed three times with 0.1 M sodium phosphate buffer (pH 7.4). The samples were then fixed with 1% osmic acid for 2 h at 25 °C and dehydrated through an ethanol gradient. For SEM, the samples were then directly examined with a Hitachi SU8100 SEM microscope (Hitachi, Tokyo, Japan) using an acceleration voltage of 3.0 kV. For TEM, the samples were embedded in Epon 812 (Nisshin EM, Tokyo, Japan) and examined with a Hitachi HT7800 TEM microscope (Hitachi, Tokyo, Japan) using an acceleration voltage of 80 kV. 

To observe apoptosis of *F. pseudograminearum*, mycelium was collected from the PDA with 1:5 YB-185 filtrate and rinsed twice with PBS. The mycelium was then stained according to the protocol of the one-step TUNEL Apoptosis Assay Kit (Beyotime, Shanghai, China). Stained mycelium was examined with an A2 Axio microscope (Carl Zeiss, Oberkochen, Germany) with fluorescence detected at 450–490 nm. In addition, nuclear morphology in fungal mycelium was observed by staining with 1 μg mL^−1^ 4-6-diamidino-2-phenylindole (DAPI) as per Domachowske et al. [20]. 

### 2.3. YB-185 Suppression of FCR in Greenhouse 

For preparation of *F. pseudograminearum* inoculum, millet seeds (cultivar: Yugu 31) were sterilized at 121 °C for 30 min in flasks and then inoculated with four 7-day-old fungal mycelial plugs (5 mm in diameter) The flasks were incubated at 25 °C for 10 days, during which the flasks were shaken once a day. Soil (sandy loam) collected from the field in Jiaozuo (Henan, China) was used for the greenhouse experiment, with the content of total N 221.6 mg/kg, available P 16.8 mg/kg, and available K 137.5 mg/kg. Then, the fungal inoculum was mixed into sterile field soil at 0.5 % (W/W). Seeds were surface-sterilized with 1% NaClO and washed with sterile water three times, and then soaked in different concentrations of YB-185 (10^6^ to 10^9^ CFU/mL) for 2 h. For control plants, seeds were soaked in sterile water. The seeds were dried overnight and sown the next day. Four seeds were planted into 200 g of *F. pseudograminearum*-inoculated soil in 250 mL plastic pots and arranged in a completely randomized design in a greenhouse maintained at 28 °C with a 16:8 h L:D photoperiod supplied by LED light and 80% RH. Each treatment had ten replicates, and the test was conducted twice. 

At 12 days post planting (dpp), segments of the root elongation zone treated or not treated with 10^9^ CFU/mL YB-185 were embedded in paraffin and cut into thin slices in cross and longitudinal sections. The samples were then co-stained with wheat-germ agglutinin-Alexa Fluor 488 conjugate (WGA-AF488) to observe hyphae of *F. pseudograminearum* and propidium iodide (PI) to observe plant cell walls [21]. The images were scanned and digitized as previous described [22]. 

At 35 dpp, plants from six pots were removed from the soil, and disease severity was graded on the washed roots using a 0-to-7 scale according to Smiley et al. [23]. Disease index and control efficiency was calculated using the formulas: 

Disease index = 100 × ∑ (grade × the number of infected plants)/(highest grade × the total number of investigated plants); 

Control efficiency = (disease index of control − disease index of treatment)/disease index of control × 100%. 

### 2.4. YB-185 Suppression of FCR in the Field 

In 2019, wheat seeds of cultivar Zhengmai 366 were soaked in 10^9^ CFU/mL YB-185 cell suspensions as described above. The seeds soaked in sterile water for 2 h were used as a non-treated control. Wheat seeds coated with 2 mL/kg difenoconazole-fludioxonil (4.8%; Syngenta, Beijing, China) were applied as a fungicide treatment control. Seeds were planted October 19 in field plots in Jiaozuo (Henan, China) that had been fertilized with 225 kg/ha carbamide and 120 kg/ha diammonium phosphate 2 days before sowing. The plots were 1.5 × 12 m and arranged in randomized complete block design with three replicates each of YB-185 treatment, fungicide treatment, and non-treated control. Plots were weeded by hand and irrigated as needed. In the pustulation growth stage of wheat, disease severity was rated according to the crown and lower stem tissues of plants on a 0-to-10 scale, where 0 = no discoloration and 10 = severe disease [24]. The control efficiency against FCR was calculated as above. In addition, grain yield was recorded at harvest time. 

### 2.5. Transcriptome of Fusarium pseudograminearum Co-Cultured with YB-185 

First, 2 mL of conidia of *F. pseudograminearum* from CMC broth (10^7^ conidia/mL) was inoculated into 50 mL PDB and incubated at 25 °C shaking at 160 rpm. At 20 h, 5 mL of YB-185 (10^8^ CFU/mL) was added and incubated at 30 °C, shaking at 160 rpm. The control was the addition of 5 mL sterile water. After 4 h and 16 h, the mycelium was filtered through two layers of sterile Miracloth (Merck Millipore, Billerica, MA, USA) and washed thoroughly with cold distilled water. Then, the samples were frozen in liquid nitrogen and stored at −80 °C. The experiment was repeated three times for each treatment. RNA extraction was done using a RNeasy Mini kit (Qiagen, Hilden, Germany), and the RNA was sent to Shanghai Meiji Biomedical Technology Company (Shanghai, China) for sequencing using an Illumina HiSeq platform. 

To obtain clean reads, raw reads in fastq format were filtered by removing adapters and low-quality reads [25]. Q score, GC content, and sequence duplication level were calculated to assess quality. For annotation, clean reads were mapped to the genome *Fusarium pseudograminearum* CS3096 (https://www.ncbi.nlm.nih.gov/genome/14399?genome_assembly_id=293398, accessed on 1 April 2022). Transcriptome assembly was accomplished by StringTie [26]. The assembled transcripts were searched against the databases Pfam (http://pfam.xfam.org/, accessed on 1 April 2022), Gene Ontology (Go) (http://www.geneontology.org/, accessed on 1 April 2022), Clusters of Orthologous Groups of proteins (COG) (http://eggnog5.embl.de/#/app/home, accessed on 1 April 2022), Kyoto Encyclopedia of Gene and Genomes (KEGG) (http://www.genome.jp/kegg/, accessed on 1 March 2022), and Swiss-Prot (https://www.expasy.org/, accessed on 1 April 2022). 

Expression levels were calculated from fragments per kb of transcript per million reads (FPKM). Differentially expressed genes (DEGs) were identified with the DESeq2 R package (1.10.1) with |log_2_(fold change)| > 1 using a false discovery rate (FDR < 0.01) and a high statistically significant value (*p* < 0.05). DEGs were divided into functional categories and defined pathways. GO enrichment analysis was implemented by the GOseq R packages [27] with a corrected *p*-value < 0.05 as the threshold. KEGG pathway analysis was performed in the KEGG database. Global metabolic pathways were displayed using iPath 2.0 (http://pathways.embl.de, accessed on 1 April 2022). 

### 2.6. RT-PCR of Fusarium pseudograminearum Genes in Co-Cultures with YB-185 

To verify the reliability of the transcriptome data of *F. pseudograminearum*, 30 DEGs were selected based on low q-value, large fold difference, and annotation (Table S1). Tubulin was used as the internal reference gene (Table S2). cDNAs were synthesized from the sequencing RNAs using a PrimeScript™ RT Reagent Kit with gDNA Eraser (TaKaRa, Dalian, China), and primers were designed using Primer 5.0 (Table S2). Reverse transcription quantitative PCR (RT-qPCR) was conducted using a Step One Plus Real-Time PCR System (Applied Biosystems, Foster City, CA, USA) with SYBR Green MasterMix (Applied Biosystems, Foster City, CA, USA). Relative expression was calculated using the 2^−ΔΔCT^ method [28] in triplicate with three biological replicates. 

### 2.7. Total Antioxidant and Glutathione-S-Transferase Activity

*Fusarium pseudograminearum* mycelia were prepared as per transcriptome sequencing described previously, and samples were collected at 4 h and 16 h. For antioxidant levels, a crude enzyme solution was prepared from the mycelia as per Han et al. [29]. The scavenging activity of 2,2′-Azino-bis(3-ethylbenzothiazoline-6-sulfonic acid) diammonium salt radical cation (ABTS·+) was determined with a Total Antioxidant Capacity (T-AOC) Assay Kit (Sangon Biotech, Shanghai, China), and activity was calculated using the equation of Han et al. [29]. Glutathione-S-transferase (GST) activity was determined from a crude enzyme solution prepared from the mycelia as per Wang et al. [30]. GST activity was determined with a Glutathione S-transferase Activity Assay Kit (Solarbio, Beijing, China). 

### 2.8. Statistical Analysis

Data were analyzed using SPSS Statistics 26.0 (IBM, Armonk, NY, USA). After ANOVA assumptions were evaluated using the Kolmogorov–Smirnov test for normality and Levene’s test for homogeneity between groups, one-way ANOVA was performed (*p* < 0.05). 

## 3. Results

### 3.1. Strain YB-185 Isolation and Identification 

**Figure 1:**
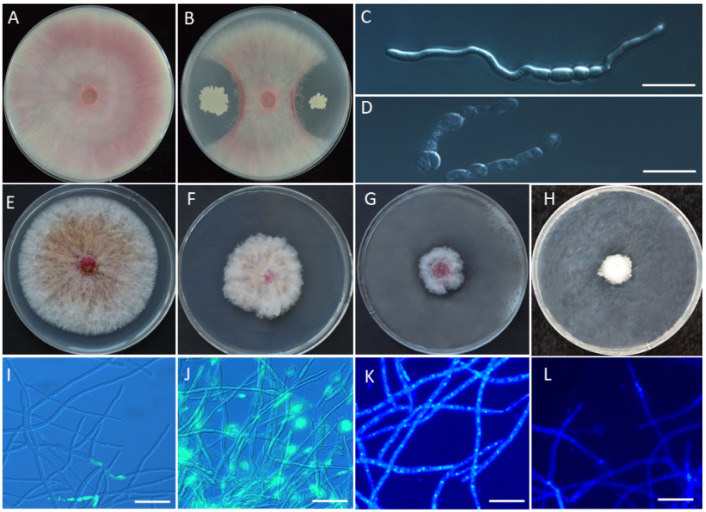
Effect of YB-185 and YB-185 culture filtrate on *Fusarium pseudograminearum*. (**A**) Pure culture of *F. pseudograminearum* on PDA; (**B**) dual-culture of *F. pseudograminearum* and YB-185 on PDA; (**C**) conidial germination of *F. pseudograminearum* in pure culture at 12 h; (**D**) conidial germination of *F. pseudograminearum* with 1:5 YB-185 culture filtrate at 12 h; (**E**) mycelia of *F. pseudograminearum* on PDA; (**F**–**H**) mycelia of *F. pseudograminearum* on PDA containing 1:10, 1:5, 1:2 YB-185 culture filtrates, respectively; (**I**) green fluorescence indicating apoptosis by TUNEL staining of *F. pseudograminearum* on PDA; (**J**) TUNEL staining of *F. pseudograminearum* on PDA containing 1:5 YB-185 culture filtrate; (**K**), fluorescence indicating nuclear fragmentation by DAPI staining of *F. pseudograminearum* on PDA; (**L**) DAPI staining of *F. pseudograminearum* on PDA containing 1:5 YB-185 culture filtrate. Bar = 10 μm.

A total of 102 bacteria were isolated from rhizosphere soil of wheat with FCR. All of them were used for growth inhibition of *F. pseudograminearum* by the dual-culture method, and the results showed that 45 bacterial isolates could inhibit growth of *F. pseudograminearum* isolate WZ-8A on PDA. The greatest antagonistic activity was with strain YB-185 (Figure 1A,B). Growth inhibition of *F. pseudograminearum* with YB-185 was 69.1%.

**Figure 2:**
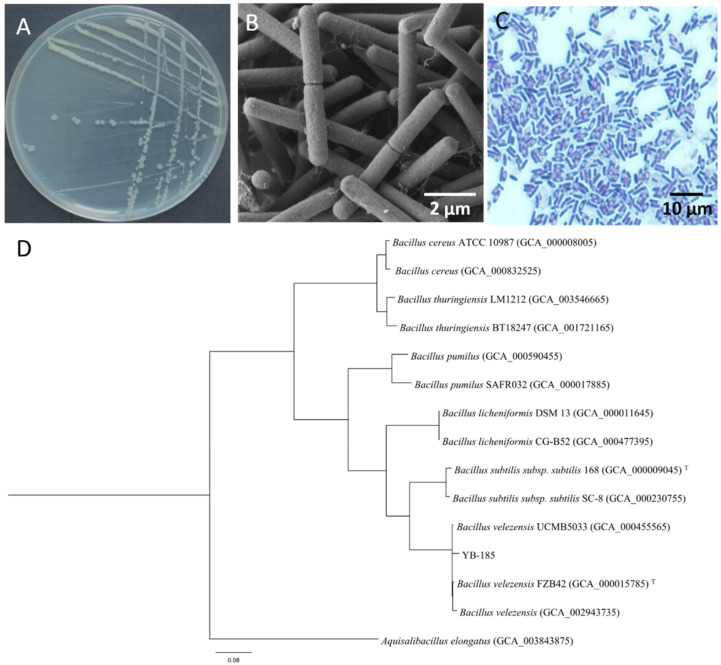
Characteristics of YB-185. (**A**) Colony morphology of YB-185 on NA at 24 h and 30 °C; (**B**) SEM of YB-185 cells (10,000×); (**C**) light microscopy of YB-185 cells and endospores with Gram staining (1600×); (**D**) maximum likelihood tree of combined 16S rRNA and gyrA sequences from YB-185 and other Bacillus spp. The tree was generated using IQ-Tree (XSEDE in the CIPRES Science Gateway) and rooted with the combined 16S rRNA and gyrA sequences of Aquisalibacillus elongatus. Scale bar shows a 0.08 genetic difference.

Colonies of YB-185 on NA were rounded, ivory white, and opaque, with irregular edges (Figure 2A). Under SEM, the cells were rod-shaped, with an average size of 3.34 μm × 0.79 μm (Figure 2B). The cells were Gram-positive, and ellipsoidal endospores were observed in the colonies (Figure 2C). The physiological parameters determined with the Biolog system showed that YB-185 was *Bacillus velezensis* with a probability of 0.936, which was consistent with the appearance of the colonies, Gram stain, and cell morphology. A phylogenetic tree based on combined 16S rRNA and *gyrA* sequences showed that YB-185 was most closely related to *B. velezensis* (Figure 2D). Based on this and the above-mentioned morphological and Biolog features, YB-185 was classified as a strain of *B. velezensis*.

### 3.2. In Vitro Antagonism of YB-185 against Fusarium pseudograminearum 

Incubation of *F. pseudograminearum* conidia with YB-185 culture filtrate resulted in most of the conidia becoming swollen and malformed (Figure 1D). Only 15.5% conidia treated with YB-185 culture filtrate had visible germ tubes at 12 h, while 95.0% conidia in the control had visible germ tubes at 12 h (Figure 1C). The inhibition percent of mycelial growth on PDA containing 1:10, 1:5, and 1:2 YB-185 culture filtrates were 44.0%, 67.9%, and 78.6%, respectively (Figure 1F–H).

**Figure 3:**
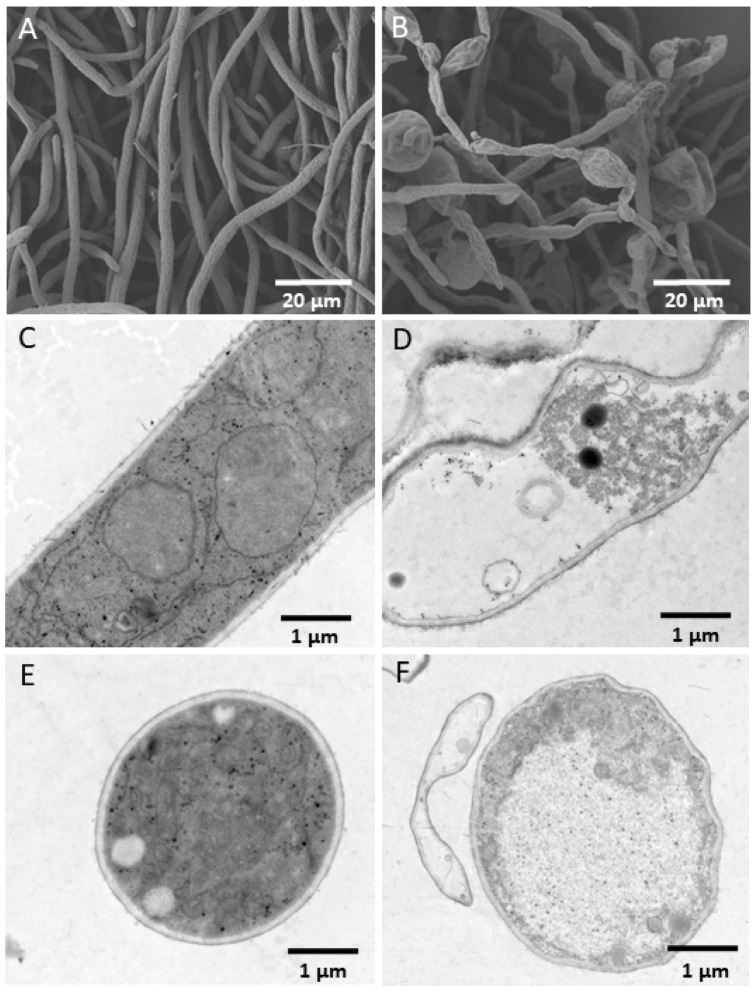
Effect of YB-185 culture filtrate on *Fusarium pseudograminearum*. (**A**) SEM of *F. pseudograminearum* hypha on PDA (1000×); (**B**) SEM of *F. pseudograminearum hypha* on PDA containing 1:5 YB-185 culture filtrate (1000×); (**C**) TEM of longitudinal section of *F. pseudograminearum* hypha on PDA (8000×); (**D**) TEM of longitudinal section of *F. pseudograminearum* hypha on PDA containing 1:5 YB-185 culture filtrate (8000×); (**E**) TEM of cross section of *F. pseudograminearum* hypha on PDA (8000×); (**F**) TEM of cross-section of *F. pseudograminearum* hypha on PDA containing 1:5 YB-185 culture filtrate (8000×).

The morphology and ultrastructure of *F. pseudograminearum* mycelia without YB-185 filtrate showed normal growth with a smooth surface (Figure 3A), while the mycelia grown with YB-185 filtrate was swollen and irregular (Figure 3B). TEM showed that the *F. pseudograminearum* hyphae without YB-185 filtrate had a smooth surface, organized cell wall, complete plasma membrane, and uniformly distributed cytoplasm and organelles (Figure 3C,E). However, hyphae grown with YB-185 filtrate had an irregular surface, degraded cell wall, broken plasma membrane, cytoplasm with discontinuously packed fibers and empty areas, and sparse and unevenly distributed organelles (Figure 3D,F). 

Using TUNEL staining, mycelia of *F. pseudograminearum* cells with YB-185 filtrate showed strong fluorescence, indicating apoptosis, mainly concentrated in the enlarged deformity at the tip of the mycelium (Figure 1J). However, the mycelia of the control showed only slight fluorescence (Figure 1I). In addition, mycelia treated with YB-185 filtrate had cells exhibiting nuclear fragmentation based on DAPI staining (Figure 1L), whereas the control mycelia did not show nuclear fragmentation (Figure 1K). 

### 3.3. YB-185 Suppression of FCR in Greenhouse

**Figure 4:**
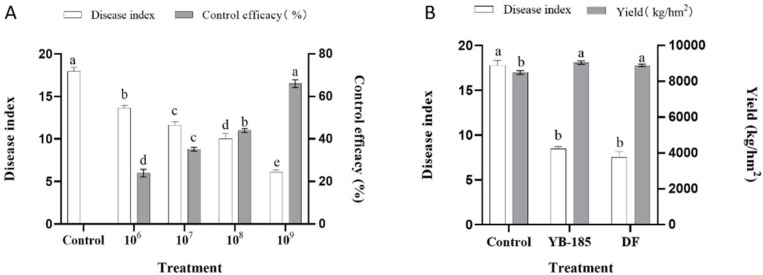
Effect of YB-185 on FCR in the greenhouse and field. (**A**) Disease index and control efficacy of FCR in the greenhouse with seed not treated (control) or treated with 10^6^, 10^7^, 10^8^, and 10^9^ CFU/mL of YB-185; (**B**) disease index and grain yield in fields infested with *F. pseudograminearum* with seed not treated (control) or seed treated with 10^9^ CFU/mL of YB-185 or 4.8% difenoconazole fludioxonil (DF). Different lowercase letters indicate a significant difference within groups (*p* < 0.05) using one-way ANOVA.

Seed treatment with 10^6^ to 10^9^ CFU/mL of YB-185 in the greenhouse showed that increasing concentration of YB-185 resulted in greater reduction in FCR (Figure S1), with the disease index (control efficiency) ranging from 13.7 (23.9%) for 10^6^ CFU/mL to 6.1 (66.1%) for 10^9^ CFU/mL of YB-185 (Figure 4A). 

**Figure 5:**
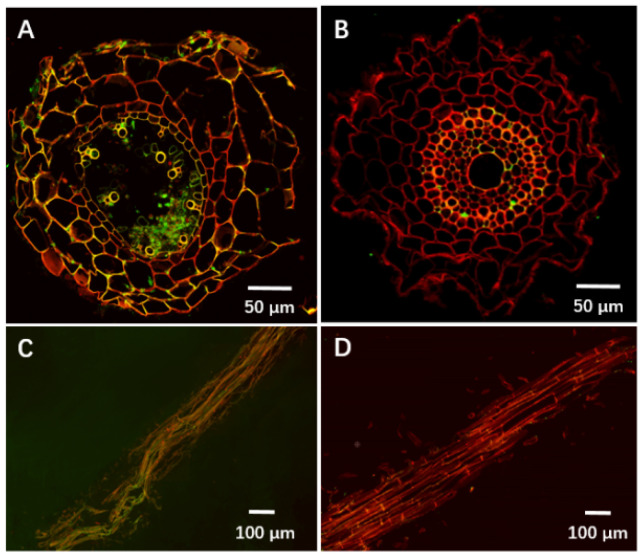
Effect of YB-185 on invasion of wheat roots by *Fusarium pseudograminearum*. (**A**) Light microscopy of cross-section of wheat root infected with *F. pseudograminearum* stained with wheat-germ agglutinin-Alexa Fluor 488 (WGA) to observe fungal hyphae by green fluorescence and propidium iodide (PI) to observe plant cell walls by red fluorescence; (**B**) cross-section of stained wheat root infected with *F. pseudograminearum* and treated with YB-185; (**C**) longitudinal section of stained wheat root infected with *F. pseudograminearum*; (**D**) longitudinal section of stained wheat root infected with *F. pseudograminearum* and treated with YB-185.

With *F. pseudograminearum* alone, hyphae were visible in the root cortex, endodermis, pericycle regions, and xylem vessels, with cells in the cortex appearing highly degraded and the endodermis cells shrunken (Figure 5A,C). However, roots infected with *F. pseudograminearum* and treated with 10^9^ CFU/mL of YB-185 showed only a limited amount of hyphae restricted to the endodermis and pericycle, with the root tissues appearing relatively intact (Figure 5B,D).

### 3.4. YB-185 Suppression of FCR in the Field

Non-treated control fields had an FCR disease index of 17.5, which was significantly higher than the 8.5 found with seed treatment of 10^9^ CFU/mL YB-185 (*p* < 0.05) (Figure 4B). The control efficiency of FCR by YB-185 in the field at 52.0% was not significantly different from 57.6% with seed treatment of 2 mL/kg 4.8% difenoconazole fludioxonil (*p* < 0.05). Grain yield with 10^9^ CFU/mL YB-185 was 9040.7 kg/hm^2^, exceeding 8475.6 kg/hm^2^ for the non-treated control. The yield with 10^9^ CFU/mL YB-185 increased by 6.7%. 

### 3.5. Transcriptome of F. pseudograminearum Co-Cultured with B. velezensis YB-185

**Figure 6:**
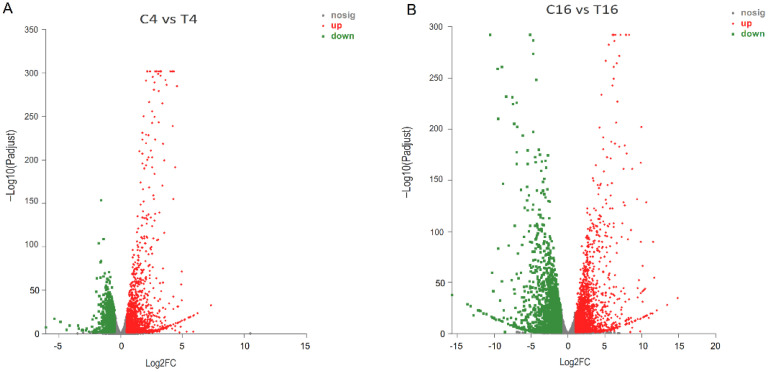
Volcano plot displaying differentially expressed genes (DEGs) in *F. pseudograminearum* co-cultured with YB-185. (**A**) DEGs in *F. pseudograminearum* co-cultured with YB-185 at 4 h; (**B**) DEGs in *F. pseudograminearum* co-cultured with YB-185 at 16 h. The y-axis corresponds to the mean expression value of log10 (*p*-value), and the x-axis displays the log2 fold change value. Red dots represent up-regulated DEGs, and green dots represent down-regulated DEGs. Gray dots indicate genes that are not differentially expressed. C4 and T4 samples are RNAs of *F. pseudograminearum* cultured for 4 h without or with YB-185, respectively, and C16 and T16 samples are RNAs of *F. pseudograminearum* cultured for 16 h without or with YB-185, respectively.

The mycelia of *F. pseudograminearum* co-cultured with YB-185 in PDB began to appear swollen and irregular at 4 h and appeared cracked and melted at 16 h. However, the mycelia of the control showed normal growth with a smooth surface at both time points (Figure S2). Sequencing RNA from *F. pseudograminearum* alone or co-cultured with YB-185 at 4 h and 16 h in PDB resulted in a total of 88.7 Gb reads after cleaning and quality check. The Q20 percentage of each library was 98.5% to 98.7%, and the Q30 percentage was 95.6% to 96.0% (Table S3). A mean of 86.8% clean reads was mapped to the *F. pseudograminearum* genome database (Table S4). The heatmap of Person’s correlation coefficients showed that the values reached more than 0.989 between repeats of each treatment (Figure S3). PCA analysis mapping also showed that the repeats of each treatment tended to aggregate together. These results showed that the replications of the transcriptomes of each treatment had a high consistency and reliability (Figure S4). Based on the FPKM mapped read and FPKM density distributions, there were differences in the dispersion and population distribution of gene expression between the treatment and control at both 4 h and 16 h (Figure S5). There were 5086 DEGs for *F. pseudograminearum* with compared to without YB-185 (|log2(fold change)| > 1, *p* < 0.05). There were 1524 and 2130 up-regulated DEGs at 4 h and 16 h, respectively, and 829 DEGs up-regulated at both time points (Figure 6). There were also 870 and 2044 down-regulated DEGs at 4 h and 16 h, respectively, and 415 DEGs down-regulated at both time points. 

All the DEGs were categorized into 45 GO terms with regard to biological process, cellular component, and molecular function (Table S5). Metabolic process (GO:0008152) was the most enriched term among biological processes, followed by cellular process (GO:0009987) and single-organism process (GO:0044699) (Figure S6). The most enriched terms among cellular components were membrane (GO:0016020), membrane part (GO:0044425), and cell (GO:0005623). The most enriched term among molecular functions were for catalytic activity (GO:0003824) and binding (GO:0005488). 

Overall, 2024 DEGs were classified into 119 KEGG pathways and categorized into metabolism, genetic information processing, cellular processes, environmental information processing, organismal systems, and human diseases (Table S6). The top 10 pathways with the most DEGs were ribosome (map03010); purine metabolism (map00230); ribosome biogenesis in eukaryotes (map03008); pyrimidine metabolism (map00240); cysteine and methionine metabolism (map00270); alanine, aspartate, and glutamate metabolism (map00250); glyoxylate and dicarboxylate metabolism (map00630); aminoacyl-tRNA biosynthesis (map00970); valine, leucine, and isoleucine degradation (map00280); and tryptophan metabolism (map00380) (Figure S7). 

**Figure 7:**
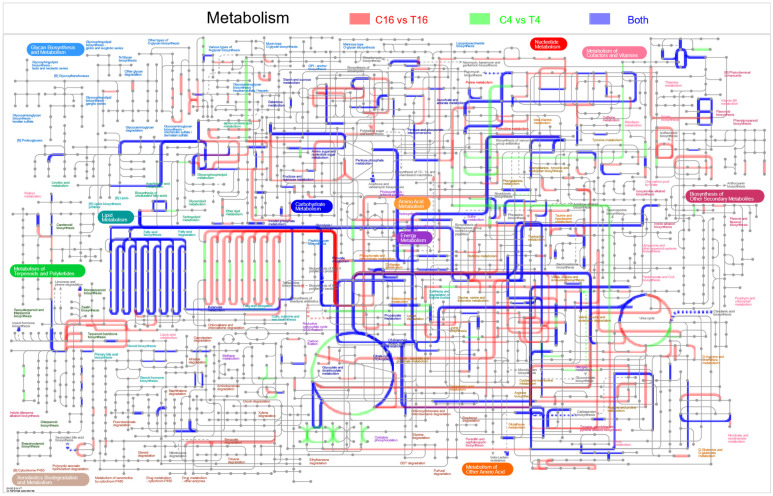
Global metabolic pathways of *F. pseudograminearum* differently expressed genes (DEGs) between pure cultures and co-cultures with YB-185. Pathways with DEGs unique to 4 and 16 h are highlighted in green and red, respectively, while DEGs at both 4 and 16 h are highlighted in blue. C4 and T4 samples are RNAs of *F. pseudograminearum* cultured for 4 h without or with YB-185, respectively, and C16 and T16 samples are RNAs of *F. pseudograminearum* cultured for 16 h without or with YB-185, respectively.

The 2024 DEGs were also classified by IPATH pathway analysis. The majority of the enriched pathways were for lipid metabolism, energy metabolism, amino acid metabolism, carbohydrate metabolism, glycan biosynthesis and metabolism, metabolism of cofactors, and vitamins (Figure 7). 

### 3.6. DEGs of Fusarium pseudograminearum Associated with Co-Cultivation with YB-185 

Related to fungal cell wall synthesis, 3-beta-glucan synthase (*FPSE_10797*) and 1,3-beta-glucanosyltransferase (*FPSE_05113*, *FPSE_03879*, *FPSE_02936*) were down-regulated at least one time point (Table 1). Chitin-synthase-related genes (*FPSE_01706*, *FPSE_09193*) were up-regulated at 4 h but not at 16 h. 

For fungal cell membrane synthesis and integrity, DEGs for ergosterol biosynthesis protein ERG3 (*FPSE_12291*) and ERG5 (*FPSE_01847*) were significantly up-regulated at both time points (Table 1). Fatty acid synthase FAS1 (*FPSE_07168*) and FAS2 (*FPSE_07169*), fatty acid elongase ELO2 (*FPSE_04157*) and ELOA (*FPSE_11763*), and mechanosensitive ion channel protein (*FPSE_03004*) DEGs were significantly down-regulated at both time points. 

Related to fungal antioxidants, DEGs for superoxide dismutase SOD1 (*FPSE_07706*), peroxiredoxin (*FPSE_10302*, *FPSE_12380* and *FPSE_11702*), and thioredoxin (*FPSE_07430* and *FPSE_05329*) were significantly down-regulated at least one time point (Table 1). However, DEGs for catalase (*FPSE_00230*), glutathione S-transferase (*FPSE_04574*, *FPSE_05333*, *FPSE_07916*, *FPSE_09673*, *FPSE_10852,* and *FPSE_01282*), ABC transporter F family member 4 (*FPSE_04311*), ABC multidrug transporter MDR (*FPSE_05712*, *FPSE_11895*), and ABC multidrug transporter B family member (*FPSE_00902*) were significantly up-regulated at both time points. Apoptosis-related DEGs for TatD-like DNase (*FPSE_05373*, *FPSE_08319*) were up-regulated at both time points (Table 1).

For fungal secondary metabolite biosynthesis, DEGs for polyketide synthetases *PKS2* were down-regulated at 4 h and up-regulated at 16 h, while *PKS6*, *PKS7,* and *PKS10* were up-regulated at 16 h. (Table 1). However, DEGs for *PKS12* were down-regulated at both time points. Nonribosomal peptide synthetases *NPS2* and *NPS6* were significantly down-regulated at 16 h. 

Among DEGs associated with enzymes potentially attacking plant cell components, DEGs for cellulose (*FPSE_06033*, *FPSE_03879*, *FPSE_01167*, *FPSE_04467*), lipase (*FPSE_10579*, *FPSE_05068*, *FPSE_07610*, *FPSE_08639*, *FPSE_08884*, *FPSE_08802*), amylase (*FPSE_01774*, *FPSE_06584*, *FPSE_05690*), endo-beta-1,4-glucanase (*FPSE_01902*), endoglucanase (*FPSE_00619)*, endo-1,4-beta-xylanase (*FPSE_05752*, *FPSE_07423*), and laccase (*FPSE_07047*, *FPSE_08810*) were all significantly down-regulated at 16 h (Table 1). However, DEGs for the plant-cell-wall-degrading enzyme pectinesterase (*FPSE_09929*) and synthesis of toxic trichothecenes (*FPSE_11049*, *FPSE_02231*, *FPSE_05694*, *FPSE_10392*, *FPSE_08720*) were significantly up-regulated at both time points. 

### 3.7. RT-PCR of Fusarium pseudograminearum Genes in Co-Cultures with YB-185

To confirm the DEG expression profiles, 36 DEGs were analyzed by qRT-PCR (Figure S8). Overall, qRT-PCR results matched well with those from RNA-seq.

### 3.8. Total Antioxidant and GST Activity

**Figure 8:**
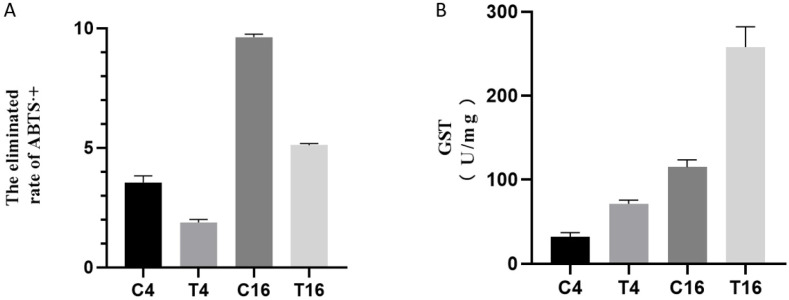
Effect of YB-185 on total antioxidant and glutathione S-transferase (GST) activity of *F. pseudograminearum*. (**A**) Free radical scavenging activity (ABTS·+ elimination rate) of *F. pseudograminearum* between pure cultures and co-cultures with YB-185; (**B**) GST activity of *F. pseudograminearum* between pure cultures and co-cultures with YB-185. C4 and T4 samples are crude protein solutions of *F. pseudograminearum* cultured for 4 h without or with YB-185, respectively, and C16 and T16 samples are crude protein solutions of *F. pseudograminearum* cultured for 16 h without or with YB-185, respectively.

Cultivation of *F. pseudograminearum* with *B. velezensis* resulted in significantly lower free radical scavenging activity as indicated by ABTS·+ clearance rates compared to the control at both 4 and 16 h (Figure 8A). In contrast, co-cultivation resulted in significantly higher GST activities at 4 h and 16 h compared to the control (Figure 8B). 

## 4. Discussion

Among reports of *Bacillus* species being used as BCAs of FCR of wheat caused by *F. pseudograminearum*, there have been *Bacillus halotolerans* [31], *B. subtilis* [12], and *B. velezensis* [32], as well as *B. velezensis* as a BCA of FCR of sorghum caused by *F. pseudograminearum* [33]. In this study, a new wheat soil bacterial strain, YB-185, was identified as *B. velezensis* and shown to be a promising BCA for FCR of wheat caused by *F. pseudograminearum*.

Seed treatment with YB-185 reduced FCR under both greenhouse and field conditions, with 10^9^ CFU/mL able to reduce disease indices by 66.1% and 52.0% in the greenhouse and field, respectively. The level of control in the field was comparable to fungicide seed treatment. Using *F. pseudograminearum* coleoptile infection, *B. halotolerans* QTH8 application to wheat seedlings decreased FCR in wheat by 62.4% [31]. Using *F. pseudograminearum* inoculated soil, *B. subtilis* YB-15 wheat seed treatment reduced the FCR by 81.5% [12], priming of wheat seeds with *B. velezensis* UTB96 reduced FCR by 65.5% [32], and priming of sorghum plants with *B. velezensis* NB54 resulted in reduced disease severity by 47.6% under drought stress and 55.6% without drought stress [33]. In general, *B. velezensis* YB-185 provided similar levels of control of FCR compared to those other *Bacillus* strains, but all those studies were limited to greenhouse conditions, whereas this study included a one-year field study to verify the potential of YB-185 as a BCA under more realistic conditions. This is important as many BCAs that perform well under controlled conditions fail to perform similarly in the field [34]. The seed treatment of 10^9^ CFU/mL YB-185 significantly decreased the disease index by 52.0% compared to control, and subsequently retrieved yield lost by 6.7%. To prevent the occurrence of FCR and reduce the losses of the yield to a great extent, the application methods of the biocontrol strain should be optimized in future work, and the biocontrol agent should be applied once again at the returning green stage. The use of *B. velezensis* YB-185 for the control of FCR under practical cultivation conditions may represent an efficient alternative to fungicides for sustainable wheat cultivation.

At least one mode of action of YB-185 against *F. pseudograminearum* was direct antimicrobial activity, as evidenced by growth inhibition in culture. This may be related to secreted compounds from YB-185, as culture filtrates were able to reduce spore germination and cause swollen and malformed hyphae and conidial germ tubes. The cytoplasm of fungal cells exposed to YB-185 culture filtrate contained empty and fiber-filled areas, and the organelles appeared sparse and unevenly distributed. A likely candidate for such activity would be antimicrobial peptides, which are well known in *Bacillus* species, especially lipopeptides, which have been involved in its biocontrol effect against many fungi [35]. Among lipopeptide studies, Liao et al. [36] reported swollen and cracked hyphae of *Pyricularia oryzae* exposed to fengycin from *B. amyloliquefaciens* BPD1, Gong et al. [37] reported that the cytoplasm of *F. graminearum* hyphae was disorganized and sparse after exposure to iturin A or plipastatin A from *B. amyloliquefaciens* S76-3, and Toral et al. [38] reported that the organelles of *Botrytis cinerea* hyphae were degenerated and gathered in clumps after exposure to a mixture of lipopeptides from *B. methylotrophicus* XT1. Thus, the effects of culture filtrate of YB-185 on *F. pseudograminearum* hyphae were similar to those previously reported for fungi exposed to different *Bacillus* lipopeptides.

In this study, the transcriptome response of *F. pseudograminearum* to YB-185 culture filtrate showed changes related to fungal cell wall and membrane synthesis, response to oxidative stress, cell death related to apoptosis, production of secondary metabolites, and factors potentially related to virulence to plants. Thus far, there are several transcriptome studies of plant pathogenic fungi incubated with *Bacillus* species or their crude culture filtrates. For example, RNA was analyzed from *Colletotrichum gloeosporioides* TS-09 grown on PDA with *B. amyloliquefaciens* SDF-005 for 9 days [39], *Verticillium dahliae* VdSHZ-9 grown on PDA with *Bacillus* N-4 for 7 days [40], and *Fusarium oxysporum* grown on PDA with *B. subtilis* HSY21 for 2 and 3 days [29]. RNA was also analyzed for *Sclerotinia* *sclerotiorum* 1980 grown on PDA containing 10% culture filtrate of *B. amyloliquefaciens* Bam22 for 1 day [41], and *Botrytis cinerea* strain B05.10 grown in PDB with *B. subtilis* MBI 600 culture filtrate for 0, 24, 48, and 72 h [42].

One effect of the YB-185 on *F. pseudograminearum* was on fungal cell wall synthesis genes. There was down-regulation of one 1,3-beta-glucan synthase and three 1,3-beta-glucanosyltransferase genes at 4 and 16 h. Note that 1,3-Beta-glucan synthase is a glucosyltransferase that generates beta-glucan, a major component of fungal cell walls, and 1,3-beta-glucanosyltransferase is involved in the elongation of 1,3-beta-glucan [43]. For both *S. sclerotiorum* and *V. dahilae* with *Bacillus*, 1,3-beta-glucan synthase DEGs were up-regulated [40,41]. It was concluded that this was an attempt to alleviate cell wall damage. In this study, there was an up-regulation of two chitin synthase genes only at 4 h, which could be an early attempt to repair cell wall injuries. Chitin synthase is a glycosyltransferase that catalyzes formation of chitin, another major component of fungal cell walls [44]. Tian et al. [40] reported that DEGs related to chitin synthase in *V. dahilae* were up-regulated by *Bacillus*. Overall, there may be more limited new cell wall synthesis of *F. pseudograminearum* with *Bacillus* stress limited to earlier in the interaction.

YB-185 also altered the expression of genes related to fungal cell membranes. There was up-regulation of two ergosterol synthase DEGs at both time points. Ergosterol is the major component of fungal cell membranes [45]. Some fungicides, such as terbinafine and naftifine, target ergosterol synthesis enzymes, resulting in fungal lysis [45]. Similarly, there was up-regulation with *B. amyloliquefaciens* stress for two ergosterol synthesis pathway-related genes in *S. sclerotiorum* [41] and *C. gloeosporioides* [39]. It was suggested that those changes promoted membrane fluidity, reducing the effects of antimicrobial substances. Two fatty acid synthases and two fatty acid elongase DEGs of *F. pseudograminearum* were down-regulated at both time points with YB-185. Fatty acids with phospholipids are major components of membranes and maintain cell membrane fluidity [46]. In contrast, two fatty acid synthesis pathway-related genes were up-regulated in *S. sclerotiorum* with *B. amyloliquefaciens* [41]. It was proposed that this was part of an attempt to reduce lipopeptide membrane damage, but this did not occur with *F. pseudograminearum*. Expression of a mechanosensitive ion channel membrane protein DEG was down-regulated at both time points. Mechanosensitive ion channels provide protection against hypoosmotic shock, responding to membrane tension, and reduced levels of them could result in membrane fragility and loosening [47]. However, other transcriptome studies of plant pathogenic fungi with *Bacillus* did not report DEGs related to mechanosensitive ion channels [29,39,40,41,42].

Another impact of YB-185 was on the antioxidative stress response of *F. pseudograminearum.* Two peroxiredoxin DEGs were down-regulated at 4 h, and one SOD, three peroxiredoxin, and two thioredoxin DEGs were down-regulated at 16 h. SODs convert the damaging free radical superoxide anion into oxygen and hydrogen peroxide [48]. Peroxiredoxins are cysteine-dependent peroxidases that limit peroxide levels within cells [49], and thioredoxins reduce oxidized cysteine residues cleaving disulfide bonds, protecting proteins from oxidative aggregation and inactivation [50]. With *Bacillus* or culture filtrate, however, *S. sclerotiorum* up-regulated peroxidase and catalase DEGs [41], and *V. dahilae* up-regulated SOD, catalase, peroxiredoxin, and thioredoxin DEGs [40]. This was proposed to be part of an ROS stress response. Evidence for an increased antioxidative stress response of *F. pseudograminearum* was up-regulation of one catalase, six GST, and four ABC transporter DEGs at both time points. Catalase breaks hydrogen peroxide into oxygen and water [51], GST can conjugate the reduced form of glutathione to xenobiotics for detoxification [52], and ABC transporter can transfer substrates across membranes to remove toxins from cells [53]. Similarly, *C. gloeosporioides, Setosphaeria turcica,* and *B. cinerea* DEGs for GSTs and/or ABC transporters were up-regulated with *Bacillus* stress [39,42,54]. However, a catalase DEG of *F. oxysporum* was down-regulated with *Bacillus* stress [29]. Thus, not all elements of fungal antioxidative stress response are up-regulated with *Bacillus* stress. Despite increased expression of *F. pseudograminearum* catalases and GSTs, the scavenging activity of ABTS·+ indicated that total antioxidant capacity was lowered, indicating YB-185-induced oxidative stress to *F. pseudograminearum*.

YB-185 stress also up-regulated two TatD-like DNase DEGs. However, other transcriptome studies of plant pathogenic fungi with *Bacillus* have not reported TatD-like DNase DEGs [29,39,40,41,42]. TatD-like DNase degrades DNA during cell apoptosis [55]. Further supporting apoptosis of *F. pseudograminearum* with YB-185 was cell death detected by TUNEL staining and nuclear fragmentation detected by DAPI staining.

Among *F. pseudograminearum* DEGs related to secondary metabolites altered by YB-185 were two PKS DEGs down-regulated at 4 and/or 16 h, and four PKS DEGs up-regulated at 16 h. Fungal PKSs synthesize polyketides, a type of lipid, that have a wide range of functions [56]. By comparison to *F. graminearum* genome, the down-regulated PKSs of *F. pseudograminearum* were involved in mycelial growth and the red mycelial pigment aurofusarin [57,58], and the up-regulated PKSs were related to synthesis of the mycotoxins fusaristatin A and fusarin C, a regulator of perithecial maturation, mycelial growth, and aurofusarin [57,58,59,60,61]. This suggests that *F. pseudograminearum* is shifting the types of polyketides produced during the *Bacillus* stress response. Two NPS DEGs were down-regulated at 4 and/or 16 h. NPSs produce non-ribosomal peptides that can act as fungal antibiotics, toxins, and siderophores [62]. The down-regulated NPS DEGs in this study are similar to those for synthesis of the iron-binding siderophores ferricrocin and fusarinine C/triacetylfusarinine C [63,64]. Those types of siderophores are virulence determinants of many ascomycetes, including *Fusarium* [65]. The down-regulation of secondary metabolites in *F. pseudograminearum* could limit its ability to invade wheat roots, contributing to the lower FCR severity with YB-185 treatment. However, other transcriptome studies of plant pathogenic fungi with *Bacillus* stress did not report DEGs related to PKS and NPS [29,39,40,41,42].

Another impact of YB-185 culture filtrate was on DEGs for cell wall-degrading enzyme (CWDE) synthesis of *F. pseudograminearum*. DEGs for four cellulases, six lipases, three amylases, one endo-beta-1,4-glucanase, one endoglucanase, two endo-1,4-beta-xylanases, and two laccases were down-regulated at 16 h. Similarly, DEGs for CWDE synthesis for amylase, glucosidase, xylanase, and cellulase of *F. oxysporum* were significantly down-regulated under the stress of *B. subtilis* [29]. It was proposed that this resulted in the reduction of virulence to the host plant. A reduced ability to degrade host tissues could explain the microscopy observations of *F. pseudograminearum*-infected root tissues remaining more intact, with hyphae being limited to the endodermis and pericycle with YB-185 treatment. However, one CWDE synthesis DEG for pectinesterase was up-regulated. Pectinesterase acts in plant cell wall modification and is a virulence factor of *Botrytis cinerea* [66].

Expression of two DEGs for trichothecene 3-O-acetyltransferase and two DEGs for trichothecene efflux pump were up-regulated with YB-185 culture filtrate. Trichothecenes are six-membered ring compounds with an epoxid or tricyclic ether, acting as protein synthesis inhibitors with toxicity to eukaryotes [67]. Trichothecenes are also virulence factors for spread invasion of *Fusarium* on spikelets [68]. However, a trichothecene 3-O-acetyltransferase can act in self-protection by converting highly toxic trichothecenes to less toxic compounds [69], and a trichothecene efflux pump can do the same by exporting trichothecene [70]. Thus, this may primarily be a self-protection mechanism in the stress response to *Bacillus*.

**Figure 9:**
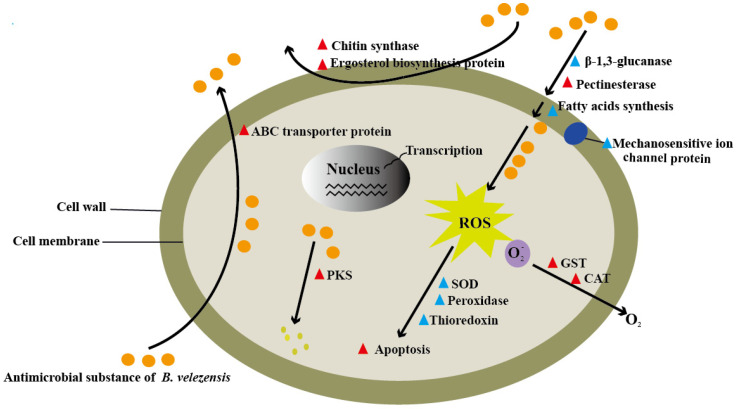
Overview of responses of *Fusarium pseudograminearum* co-cultured with *Bacillus velezensis.* Red triangles represent up-regulated DEGs, and blue triangles represent down-regulated DEGs.

In summary, the soil bacterium, *B. velezensis* YB-185 was a strong inhibitor of *F. pseudograminearum* growth both in vitro and in vivo resulting in significant control of FCR under both controlled and field conditions. An examination of *F. pseudograminearum* exposed to *B. velezensis* or its culture filtrate showed damage to cell walls and membranes, reduced antioxidant defenses, and apoptosis. Transcriptome analysis showed that the fungus can respond and attempt to alleviate damage caused by *B. velezensis*. These include trying to increase cell wall and membrane synthesis, antioxidant responses, detoxification, and export of xenobiotics from its cells (Figure 9). However, these were insufficient to prevent cell damage, and its ability to cause FCR was greatly compromised with YB-185.

## Figures and Tables

**Table 1:** Fold changes in *F. pseudograminearum* differently expressed genes (DEGs) between pure cultures and co-cultures with YB-185 at 4 h and 16 h cultivation. Sequence ID is the assembled unigene; gene name is from the mapped gene in the genome of *Fusarium pseudograminearum* CS3096; and putative function from the Pfam, Swiss-Prot, and COG databases. C4 and T4 samples are RNAs of *F. pseudograminearum* cultured for 4 h without or with YB-185, respectively, and C16 and T16 samples are RNAs of *F. pseudograminearum* cultured for 16 h without or with YB-185, respectively.

Sequence ID	Gene Name	Putative Function	log2 FC(T4/C4)	log2 FC(T16/C16)
Cell wall synthesis				
gene11337	*FPSE_10797*	1,3-beta-glucan synthase	−0.98	−1.36 *
gene2172	*FPSE_05113*	1,3-beta-glucanosyltransferase ARB_07487	−1.43 *	−1.02 *
gene3142	*FPSE_03879*	1,3-beta-glucanosyltransferase gel1	−1.38 *	−2.32 *
gene8431	*FPSE_02936*	1,3-beta-glucanosyltransferase gel2	0.03	−1.90 *
gene9847	*FPSE_01706*	chitin synthase 6	3.60 *	0.62
gene2063	*FPSE_09193*	chitin synthase 8	1.06 *	−0.24
Cell membrane synthesis				
gene7320	*FPSE_12291*	ergosterol biosynthesis protein ERG3	1.26 *	1.56 *
gene5907	*FPSE_01847*	ergosterol biosynthesis protein ERG5	2.85 *	2.93 *
gene7662	*FPSE_07169*	fatty acid synthase subunit alpha FAS2	−2.35 *	−3.55 *
gene7663	*FPSE_07168*	fatty acid synthase subunit beta FAS1	−2.18 *	−4.06 *
gene10219	*FPSE_11763*	fatty acid elongase (ELOA)	−1.01 *	−2.14 *
gene10703	*FPSE_04157*	fatty acid elongase (ELO2)	−1.33 *	−1.07 *
gene8365	*FPSE_03004*	mechanosensitive ion channel protein Msy1	−1.17 *	−1.25 *
Antioxidants				
gene4661	*FPSE_07706*	*SOD1*	−0.27	−1.15 *
gene10897	*FPSE_10302*	peroxiredoxin PRX1	−2.36 *	−2.33 *
gene3471	*FPSE_12380*	peroxiredoxin DOT5	−1.28 *	−2.21 *
gene4615	*FPSE_11702*	peroxiredoxin	−0.26	−1.24 *
gene3597	*FPSE_07430*	thioredoxin	−0.71	−4.21 *
gene6188	*FPSE_05329*	thioredoxin	−0.7	−2.34 *
gene2370	*FPSE_00230*	catalase	5.85 *	4.56 *
gene6030	*FPSE_04574*	glutathione S-transferase	2.12 *	3.90 *
gene6184	*FPSE_05333*	glutathione S-transferase	3.79 *	3.99 *
gene11189	*FPSE_07916*	glutathione S-transferase-like protein FUS3	2.99 *	5.34 *
gene12001	*FPSE_09673*	Glutathione S-transferase	3.33 *	2.36 *
gene6812	*FPSE_10852*	glutathione S-transferase-like protein OpS6	1.01 *	1.16 *
gene780	*FPSE_01282*	glutathione S-transferase-like protein ustS	1.02 *	1.79 *
gene11533	*FPSE_04311*	ABC transporter F family member 4	6.37 *	6.92 *
gene3848	*FPSE_05712*	ABC multidrug transporter MDR2	1.73 *	1.91 *
gene10087	*FPSE_11895*	ABC multidrug transporter mdr1	2.95 *	3.54 *
gene8542	*FPSE_00902*	ABC multidrug transporter B	1.41 *	1.93 *
Apoptosis				
gene6144	*FPSE_05373*	TatD_DNase	9.93 *	11.2 *
gene8111	*FPSE_08319*	TatD_DNase	2.03 *	-
Secondary metabolites				
gene2473	*PKS2*	highly reducing polyketide synthase azaB	−1.57 *	3.38 *
gene2057	*PKS6*	highly reducing polyketide synthase 40	4.28 *	9.86 *
gene4735	*PKS7*	reducing polyketide synthase FUB1	1.06 *	2.32 *
gene11187	*PKS10*	polyketide synthase dehydratase	−0.07	6.02 *
gene2491	*PKS12*	non-reducing polyketide synthase PKS12	−1.75 *	−4.57 *
gene7713	*NPS2*	nonribosomal peptide synthetase 2	0.61	−2.45 *
gene5972	*NPS6*	nonribosomal peptide synthetase 6	−1.40 *	−4.50 *
Virulence				
gene9294	*FPSE_04467*	cellulase/esterase CelE	−0.24	−3.48 *
gene8741	*FPSE_01167*	cellulase (glycosyl hydrolase family 5)	−0.27	−1.67 *
gene4558	*FPSE_06033*	cellulase (glycosyl hydrolase family 5)	−2.36 *	−1.41 *
gene3142	*FPSE_03879*	cellulase (glycosyl hydrolase family 5)	−1.38 *	−2.32 *
gene10918	*FPSE_10579*	lipase 4	0.27	−1.15 *
gene11906	*FPSE_05068*	lipase_GDSL_2	−2.4 *	−1.89 *
gene1293	*FPSE_07610*	lipase_3	0.11	−2.22 *
gene2706	*FPSE_08639*	lipase_3	0.28	−1.51 *
gene4905	*FPSE_08884*	lipase_3	0.26	−1.21 *
gene9418	*FPSE_08802*	lipase_GDSL_2	−1.02	−4.36 *
gene1287	*FPSE_01774*	alpha-amylase	−0.88	−1.25 *
gene6132	*FPSE_06584*	alpha-amylase	−0.45	−6.94 *
gene3826	*FPSE_05690*	alpha-amylase	−1.37 *	−3.29 *
gene5852	*FPSE_01902*	endo-beta-1,4-glucanase D	0.54	−5.95 *
gene2343	*FPSE_00619*	endoglucanase-4	−0.38	−2.26 *
gene2125	*FPSE_05752*	endo-1,4-beta-xylanase 4	0.13	−3.14 *
gene3590	*FPSE_07423*	endo-1,4-beta-xylanase 1	−1.19 *	−0.85
gene9426	*FPSE_08810*	laccase-1 (Multicopper oxidase)	-	−4.87 *
gene11048	*FPSE_07047*	laccase	−1.64 *	−1.54 *
gene6701	*FPSE_09929*	pectinesterase	1.33 *	1.12 *
gene11282	*FPSE_11049*	trichothecene 3-O-acetyltransferase *TRI101*	6.44 *	1.10 *
gene5181	*FPSE_02231*	trichothecene 3-O-acetyltransferase *TRI101*	2.12 *	3.10 *
gene3830	*FPSE_05694*	fungal trichothecene efflux pump (*TRI12*)	4.52 *	3.79 *
gene6437	*FPSE_10392*	fungal trichothecene efflux pump (*TRI12*)	5.25 *	5.67 *
gene6518	*FPSE_08720*	fungal trichothecene efflux pump (*TRI12*)	2.35 *	5.90 *

Note: “*” represents significant difference at *p* < 0.05, “-” represents no test.

## Data Availability

The RNA-seq data presented in this study are deposited in the National Center for Biotechnology Information (NCBI). The website link is https://www.ncbi.nlm.nih.gov/sra/PRJNA776550 (accessed on 31 October 2021).

## Supplementary Materials

The following supporting information can be downloaded at: https://www.mdpi.com/article/10.3390/jof8080763/s1, Figure S1: Images of of wheat crown rot in the greenhouse with seed not treated (control) or treated with 10^6^ to 10^9^ CFU/mL of YB-185; Figure S2: Mycelia morphology of *F. pseudograminearum* co-cultured with YB-185. (A) Mycelia of *F. pseudograminearum* grown on PDA alone at 4 h, (B) Mycelia of *F. pseudograminearum* grown on PDA alone at 16 h, (C) Mycelia of *F. pseudograminearum* co-cultured with YB-185 on PDA at 4 h, (D) Mycelia of *F. pseudograminearum* co-cultured with YB-185 on PDA at 16 h. Figure S3: Heatmap of Person’s correlation coefficients between samples; Figure S4: Principal component analysis of the RNA-seq data of *F. pseudograminearum* co-cultured with *Bacillus velezensis* YB-185; Figure S5: Gene expression distributions of *F. pseudograminearum* co-cultured with *Bacillus velezensis* YB-185. (A) The violin plot showing the overall expression levels of samples. The y-axis corresponds to samples’ log10 (FPKM), (B) FPKM density distribution of samples. The y-axis corresponds to gene density, and the x-axis displays the log10 (FPKM) of samples; Figure S6: Gene ontology classification of DEGs in *F. pseudograminearum* in response to *B. velezensis* YB-185; Figure S7: KEGG enrichment analysis of DEGs in *F. pseudograminearum* in response to *B. velezensis* YB-185; Figure S8: RT-PCR validation of selected genes sampled at 4 h and 16 h. Error bars shows ± SD among the biological triplicates. Table S1: The gene information verified by RT-PCR; Table S2: The gene-specific primers used for RT-RCR; Table S3: Summary of RNA-seq data sets; Table S4: Results of clean reads mapping to *F. pseudograminearum* genome database; Table S5: Summary of GO analysis of all DEGs; Table S6: Summary of KEGG pathways analysis of all DEGs.

## References

[1] Mastrangelo A.A., Cattivelli L. (2021). What makes bread and durum wheat different?. Trends Plant Sci..

[2] Kazan K., Gardiner D.M. (2018). Fusarium crown rot caused by *Fusarium pseudograminearum* in cereal crops: Recent progress and future prospects. Mol. Plant Pathol..

[3] Murray G.M., Brennan J.P. (2010). Estimating disease losses to the Australian wheat industry. Australas. Plant Pathol..

[4] Mudge A.M., Ruth D., Yanhong D., Gardiner D.M., White R.G., Manners J.M. (2006). A role for the mycotoxin deoxynivalenol in stem colonisation during crown rot disease of wheat caused by *Fusarium graminearum* and *Fusarium pseudograminearum*. Physiol. Mol. Plant Pathol..

[5] Dyer A.T., Johnston R.H., Hogg A.C., Johnston J.A. (2009). Comparison of pathogenicity of the fusarium crown rot (FCR) complex (*F. culmorum*, *F. pseudograminearum* and *F. graminearum*) on hard red spring and durum wheat. Eur. J. Plant Pathol..

[6] Li H.L., Yuan H.X., Fu B., Xing X.P., Tang W.H. (2012). First report of *Fusarium pseudograminearum* causing crown rot of wheat in Henan, China. Plant Dis..

[7] Zhou H., He X., Wang S., Ma Q., Sun B. (2019). Diversity of the *Fusarium* pathogens associated with crown rot in the Huanghuai wheat-growing region of China. Environ. Microbiol..

[8] Blyuss K.B., Fatehi F., Tsygankova V.A., Biliavska L.O., Iutynska G.O., Yemets A.I., Blume Y.B. (2019). RNAi-based biocontrol of wheat nematodes using natural poly-component biostimulants. Front. Plant Sci..

[9] Obanor F., Neate S., Simpfendorfer S., Sabburg R., Chakraborty S. (2013). *Fusarium graminearum* and *Fusarium pseudograminearum* caused the 2010 head blight epidemics in Australia. Plant Pathol..

[10] Cheng X., Ji X., Ge Y., Li J., Qi W., Qiao K. (2019). Characterization of antagonistic *Bacillus methylotrophicus* isolated from rhizosphere and its biocontrol effects on maize stalk rot. Phytopathology.

[11] Huang Y., Wong P. (1998). Effect of *Burkholderia (Pseudomonas) cepacia* and soil type on the control of crown rot in wheat. Plant Soil.

[12] Xu W., Yang Q., Xie X., Goodwin P.H., Deng X., Zhang J., Sun R.H., Wang Q., Xia M.C., Wu C. (2022). Genomic and phenotypic insights into the potential of *Bacillus subtilis* YB-15 isolated from rhizosphere to biocontrol against crown rot and promote growth of wheat. Biology.

[13] Moya-Elizondo E.A., Jacobsen B.J. (2016). Integrated management of Fusarium crown rot of wheat using fungicide seed treatment, cultivar resistance, and induction of systemic acquired resistance (SAR). Biol. Control..

[14] Ji S.H., Paul N.C., Deng J.X., Kim Y.S., Yu S.H. (2013). Biocontrol activity of *Bacillus amyloliquefaciens* CNU114001 against fungal plant diseases. Mycobiology.

[15] Smith A.C., Hussey M.A. (2005). Gram stain protocols. ACM Microbelibrary-Laboratory Protocols.

[16] Marchesi J.R., Sato T., Weightman J.A., Martin T.A., Fry J.C., Hiom S.J., Wade W.J. (1998). Design and evaluation of useful bacterium-specific PCR primers that amplify genes coding for bacterial 16S rRNA. Appl. Environ. Microbiol..

[17] Chun J., Bae K.S. (2000). Phylogenetic analysis of *Bacillus subtilis* and related taxa based on partial *gyrA* gene sequences. Antonie Van Leeuwenhoek.

[18] Yun Y., Liu Z., Yin Y., Jiang J., Chen Y., Xu J.R., Ma Z. (2015). Functional analysis of the *Fusarium graminearum* phosphatome. New Phytol..

[19] Akpinar I., Unal M., Sar T. (2021). Potential antifungal effects of silver nanoparticles (AgNPs) of different sizes against phytopathogenic *Fusarium oxysporum* f. sp. *radicis-lycopersici* (FORL) strains. SN Appl. Sci..

[20] Domachowske J.B., Bonville C.A., Mortelliti A.J., Colella C.B., Kim U., Rosenberg H.F. (2000). Respiratory syncytial virus infection induces expression of the anti-apoptosis gene *IEX-1L* in human respiratory epithelial cells. J. Infect. Dis..

[21] Redkar A., Jaeger E., Doehlemann G. (2018). Visualization of growth and morphology of fungal hyphae in planta using WGA-AF488 and propidium iodide co-staining. Bio-Protocl.

[22] Zhang J., Yan H.X., Xia M.C., Han X.Y., Xie L.H., Paul H.G., Quan X., Sun R.H., Wu C., Yang L.R. (2020). Wheat root transcriptional responses against *Gaeumannomyces graminis* var. tritic. Phytopathol. Res..

[23] Smiley R.W., Gourlie J.A., Easley S.A., Patterson L.M. (2005). Pathogenicity of fungi associated with the wheat crown rot complex in Oregon and Washington. Plant Dis..

[24] Poole G.J., Smiley R.W., Paulitz T.C., Walker C.A., Carter A.H., See D.R., Garland-Campbell K. (2012). Identification of quantitative trait loci (QTL) for resistance to Fusarium crown rot (*Fusarium pseudograminearum*) in multiple assay environments in the Pacific Northwestern US. Theor. Appl. Genet..

[25] Lindgreen S. (2012). Adapter removal: Easy cleaning of next-generation sequencing reads. BMC Res. Notes.

[26] Pertea M., Pertea G.M., Antonescu C.M., Chang T.C., Mendell J.T., Salzberg S.L. (2015). Stringtie enables improved reconstruction of a transcriptome from RNA-seq reads. Nat. Biotechnol..

[27] Young M.D., Wakefield M.J., Smyth G.K., Oshlack A. (2010). Gene ontology analysis for RNA-seq: Accounting for selection bias. Genome Biol..

[28] Livak K.J., Schmittgen T.D. (2001). Analysis of relative gene expression data using real-time quantitative PCR. Meth.

[29] Han S., Chen J., Zhao Y., Cai H., Guo C. (2021). *Bacillus subtilis* HSY21 can reduce soybean root rot and inhibit the expression of genes related to the pathogenicity of *Fusarium oxysporum*. Pestic. Biochem. Phys..

[30] Wang J., Ma H., Zhao S., Huang J., Yang Y., Tabashnik B.E., Wu Y. (2020). Functional redundancy of two ABC transporter proteins in mediating toxicity of *Bacillus thuringiensis* to cotton bollworm. PLoS Pathog..

[31] Li S., Xu J., Fu L., Xu G., Lin X., Qiao J., Xia Y. (2022). Biocontrol of wheat crown rot using *Bacillus halotolerans* QTH8. Pathogens.

[32] Sasani M., Ahmadzadeh M., Jahansuz M.R., Navid S. (2021). Bioprimee of seed with *Bacillus velezensis* UTB96 to control the fungal pathogen of root and crown rot (*Fusarium pseudograminearum*) and improving some growth indicators of wheat. Iran. J. Seed Sci. Technol..

[33] Carlson R., Tugizimana F., Steenkamp P.A., Dubery I.A., Hassen A.I., Labuschagne N. (2020). Rhizobacteria-induced systemic resilience in *Sorghum bicolor* L. moench against *Fusarium pseudograminearum* crown rot under drought stress conditions. Biol. Control.

[34] Heydari A., Pessarakli M. (2010). A review on biological control of fungal plant pathogens using microbial antagonists. J. Biol. Sci..

[35] Ongena M., Jacques P. (2008). *Bacillus* lipopeptides: Versatile weapons for plant disease biocontrol. Trends Microbiol..

[36] Liao J.H., Chen P.Y., Yang Y.L., Kan S.C., Hsieh F.C., Liu Y.C. (2016). Clarification of the antagonistic effect of the lipopeptides produced by *Bacillus amyloliquefaciens* BPD1 against *Pyricularia oryzae* via In Situ MALDI-TOF IMS Analysis. Molecules.

[37] Gong A.D., Li H.P., Yuan Q.S., Song X.S., Yao W., He W.J., Zhang J.B., Liao Y.C. (2015). Antagonistic mechanism of iturin A and plipastatin A from *Bacillus amyloliquefaciens* S76-3 from wheat spikes against *Fusarium graminearum*. PLoS ONE.

[38] Toral L., Rodríguez M., Béjar V., Sampedro I. (2018). Antifungal activity of lipopeptides from *Bacillus* XT1 CECT 8661 against *Botrytis cinerea*. Front. Microbiol..

[39] Wang Q.H., Ji Y.P., Qu Y.Y., Qi Y.K., Li D.W., Liu Z.Y., Wu X.Q. (2020). The response strategies of *Colletotrichum gloeosporioides* s.s. due to the stress caused by biological control agent *Bacillus amyloliquefaciens* deciphered by transcriptome analyses. Biol. Control.

[40] Tian W.H., Cheng Z.R., Wang J.X., Cheng F.F., Li L.P., Huo C.X., Li W.X., Han S.Y., Guo X.Y., Wang A.Y. (2021). A transcriptome profile reveals the regulatory mechanism of *Verticillium dahliae* against *Bacillus*. Res. Sq..

[41] Yang X., Zhang L., Xiang Y., Du L., Huang X., Liu Y. (2020). Comparative transcriptome analysis of *Sclerotinia sclerotiorum* revealed its response mechanisms to the biological control agent, *Bacillus amyloliquefaciens*. Sci. Rep..

[42] Samaras A., Karaoglanidis G.S., Tzelepis G. (2021). Insights into the multitrophic interactions between the biocontrol agent *Bacillus subtilis* MBI 600, the pathogen *Botrytis cinerea* and their plant host. Microbiol. Res..

[43] Ruiz-Herrera J., Ortiz-Castellanos L. (2019). Cell wall glucans of fungi. A review. Cell Surf..

[44] Lenardon M.D., Munro C.A., Gow N.A. (2010). Chitin synthesis and fungal pathogenesis. Curr. Opin. Microbiol..

[45] Sant D.G., Tupe S.G., Ramana C.V., Deshpande M.V. (2016). Fungal cell membrane-promising drug target for antifungal therapy. J. Appl. Microbiol..

[46] Ferreri C., Masi A., Sansone A., Giacometti G., Larocca A.V., Menounou G., Scanferlato R., Tortorella S., Rota D., Conti M. (2016). Fatty acids in membranes as homeostatic, metabolic and nutritional biomarkers: Recent advancements in analytics and diagnostics. Diagnostics.

[47] Hamilton E.S., Schlegel A.M., Haswell E.S. (2015). United in diversity: Mechanosensitive ion channels in plants. Ann. Rev. Plant Biol..

[48] Wang Y., Branicky R., Noë A., Hekimi S. (2018). Superoxide dismutases: Dual roles in controlling ROS damage and regulating ROS signaling. J. Cell Biol..

[49] Perkins A., Nelson K.J., Parsonage D., Poole L.B., Karplus P.A. (2015). Peroxiredoxins: Guardians against oxidative stress and modulators of peroxide signaling. Trends Biochem. Sci..

[50] Collet J.F., Messens J. (2010). Structure, function, and mechanism of thioredoxin proteins. Antioxid. Redox Signal..

[51] Valenzuela-Cota D.F., Buitimea-Cantúa G.V., Plascencia-Jatomea M., Cinco-Moroyoqui F.J., Martínez-Higuera A.A., Rosas-Burgos E.C. (2019). Inhibition of the antioxidant activity of catalase and superoxide dismutase from *Fusarium verticillioides* exposed to a Jacquinia macrocarpa antifungal fraction. J. Environ. Sci. Health B.

[52] Ramsay E.E., Dilda P.J. (2014). Glutathione S-conjugates as prodrugs to target drug-resistant tumors. Front. Pharmacol..

[53] Rees D.C., Johnson E., Lewinson O. (2009). ABC transporters: The power to change. Nat. Rev. Mol. Cell Biol..

[54] Tian X.L., Zhang K., Wang G.L., Liu W.D. (2016). Comparative transcriptome analysis of *Setosphaeria turcica* revealed its responses mechanisms to the biological control agent *Bacillus amyloliquefaciens*. Sci. Sin. Vitae.

[55] Chang Z., Jiang N., Zhang Y., Lu H., Yin J., Wahlgren M., Chen Q. (2016). The TatD-like DNase of *Plasmodium* is a virulence factor and a potential malaria vaccine candidate. Nat. Commun..

[56] Geng Z., Zhu W., Su H., Zhao Y., Zhang K.Q., Yang J. (2014). Recent advances in genes involved in secondary metabolite synthesis, hyphal development, energy metabolism and pathogenicity in *Fusarium graminearum* (teleomorph *Gibberella zeae*). Biotechnol. Adv..

[57] Gaffoor I., Brown D.W., Plattner R., Proctor R.H., Qi W., Trail F. (2005). Functional analysis of the polyketide synthase genes in the filamentous fungus *Gibberella zeae*(anamorph *Fusarium graminearum*). Eukaryot. Cell.

[58] Frandsen R.J.N., Nielsen N.J., Maolanon N., Sørensen J.C., Olsson S., Nielsen J., Giese H. (2006). The biosynthetic pathway for aurofusarin in *Fusarium graminearum* reveals a close link between the naphthoquinones and naphthopyrones. Mol. Microbiol..

[59] Sørensen J.L., Sondergaard T.E., Covarelli L., Fuertes P.R., Giese H. (2014). Identification of the biosynthetic gene clusters for the lipopeptides fusaristatin a and W493 B in *Fusarium graminearum* and *F. pseudograminearum*. J. Nat. Prod..

[60] Song Z., Cox R.J., Lazarus C.M., Simpson T.J. (2004). Fusarin C biosynthesis in *Fusarium moniliforme* and *Fusarium venenatum*. Chembiochem.

[61] Kim D.W., Shin Y.K., Lee S.W., Wimonmuang K., Kang K.B., Lee Y.S., Yun S.H. (2021). FgPKS7 is an essential player in mating-type-mediated regulatory pathway required for completing sexual cycle in *Fusarium graminearum*. Environ. Microbiol..

[62] Martínez-Núñez M.A., López V.E.L.y. (2016). Nonribosomal peptides synthetases and their applications in industry. Sustain. Chem. Process..

[63] Oide S., Berthiller F., Wiesenberger G., Adam G., Turgeon B.G. (2015). Individual and combined roles of malonichrome, ferricrocin, and TAFC siderophores in *Fusarium graminearum* pathogenic and sexual development. Front. Microbiol..

[64] Hansen F.T., Sorensen J.L., Giese H., Sondergaard T.E., Frandsen R.J.N. (2012). Quick guide to polyketide synthase and nonribosomal synthetase genes in Fusarium. Int. J. Food Microbiol..

[65] Oide S., Moeder W., Krasnoff S., Gibson D., Haas H., Yoshioka K., Turgeon B.G. (2006). *NPS6*, encoding a nonribosomal peptide synthetase involved in siderophore-mediated iron metabolism, is a conserved virulence determinant of plant pathogenic ascomycetes. Plant Cell.

[66] Valette-Collet O., Cimerman A., Reignault P., Levis C., Boccara M. (2003). Disruption of *Botrytis cinerea* pectin methylesterase gene *Bcpme1* reduces virulence on several host plants. Mol. Plant-Microbe Interact..

[67] McCormick S.P., Stanley A.M., Stover N.A., Alexander N.J. (2011). Trichothecenes: From simple to complex mycotoxins. Toxins.

[68] Maier F.J., Miedaner T., Hadeler B., Felk A., Salomon S., Lemmens M., Kassner H., Schäfer W. (2006). Involvement of trichothecenes in fusarioses of wheat, barley and maize evaluated by gene disruption of the trichodiene synthase (*Tri5*) gene in three field isolates of different chemotype and virulence. Mol. Plant Pathol..

[69] Kimura M., Kaneko I., Komiyama M., Takatsuki A., Koshino H., Yoneyama K., Yamaguchi I. (1998). Trichothecene 3-O-acetyltransferase protects both the producing organism and transformed yeast from related mycotoxins. J. Biol. Chem..

[70] Alexander N.J., McCormick S.P., Hohn T.M. (1999). TRI12, a trichothecene efflux pump from *Fusarium* sporotrichioides: Gene isolation and expression in yeast. Mol. Gen. Genet..

