# Supplementary material for: Response of Fusarium pseudograminearum to Biocontrol Agent Bacillus velezensis YB-185 by Phenotypic and Transcriptome Analysis
Source: J Fungi (Basel). 2022 Jul 22;8(8):763. doi: 10.3390/jof8080763 (PMC9331925; doi:10.3390/jof8080763)
Supplement: Supplementary file 1 [file jof-08-00763-s001.zip › Supplementary Materials-figure.pptx]

## Slide 1
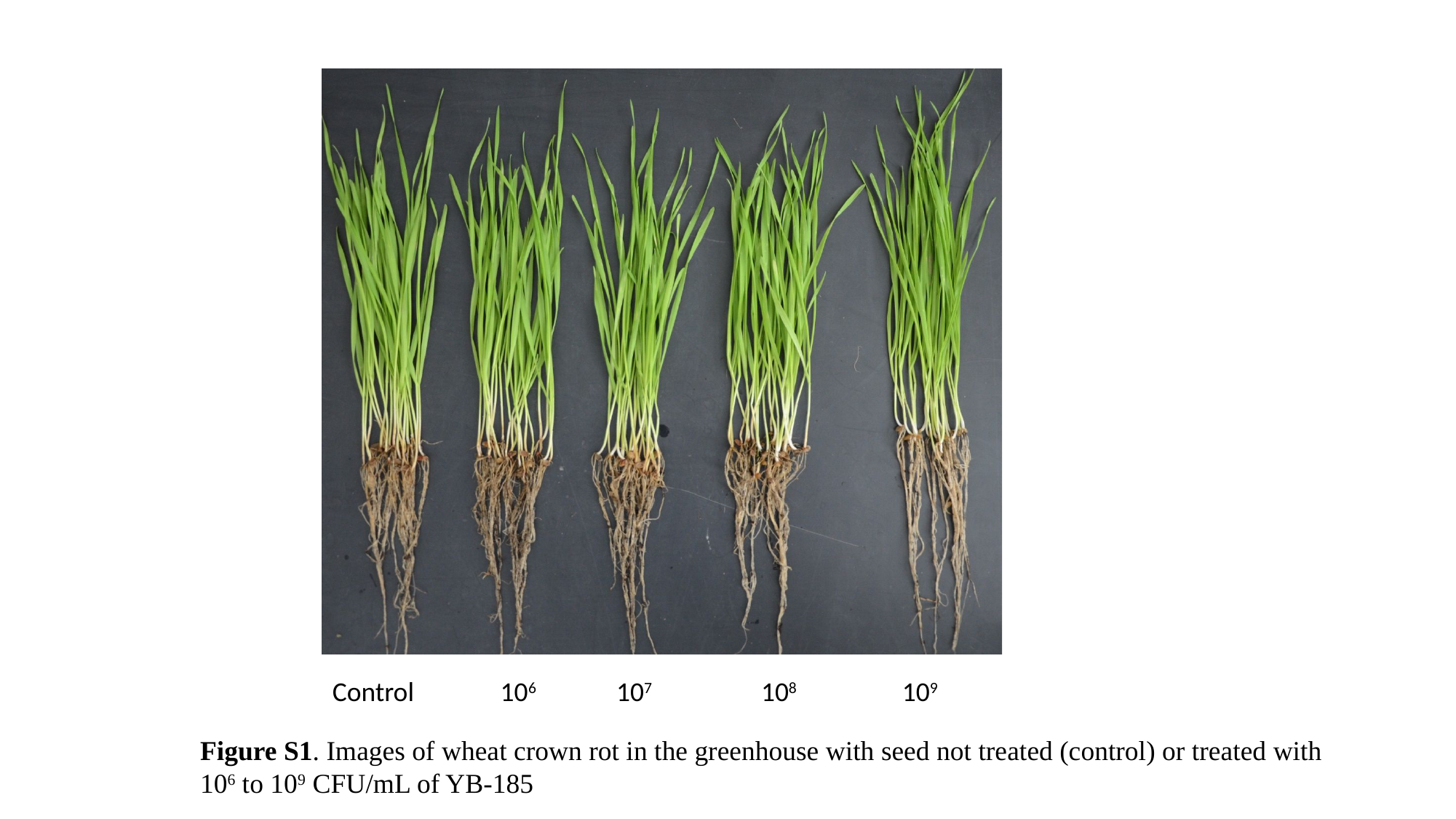

Control 106 107 108 109
Figure S1. Images of wheat crown rot in the greenhouse with seed not treated (control) or treated with 106 to 109 CFU/mL of YB-185

## Slide 2
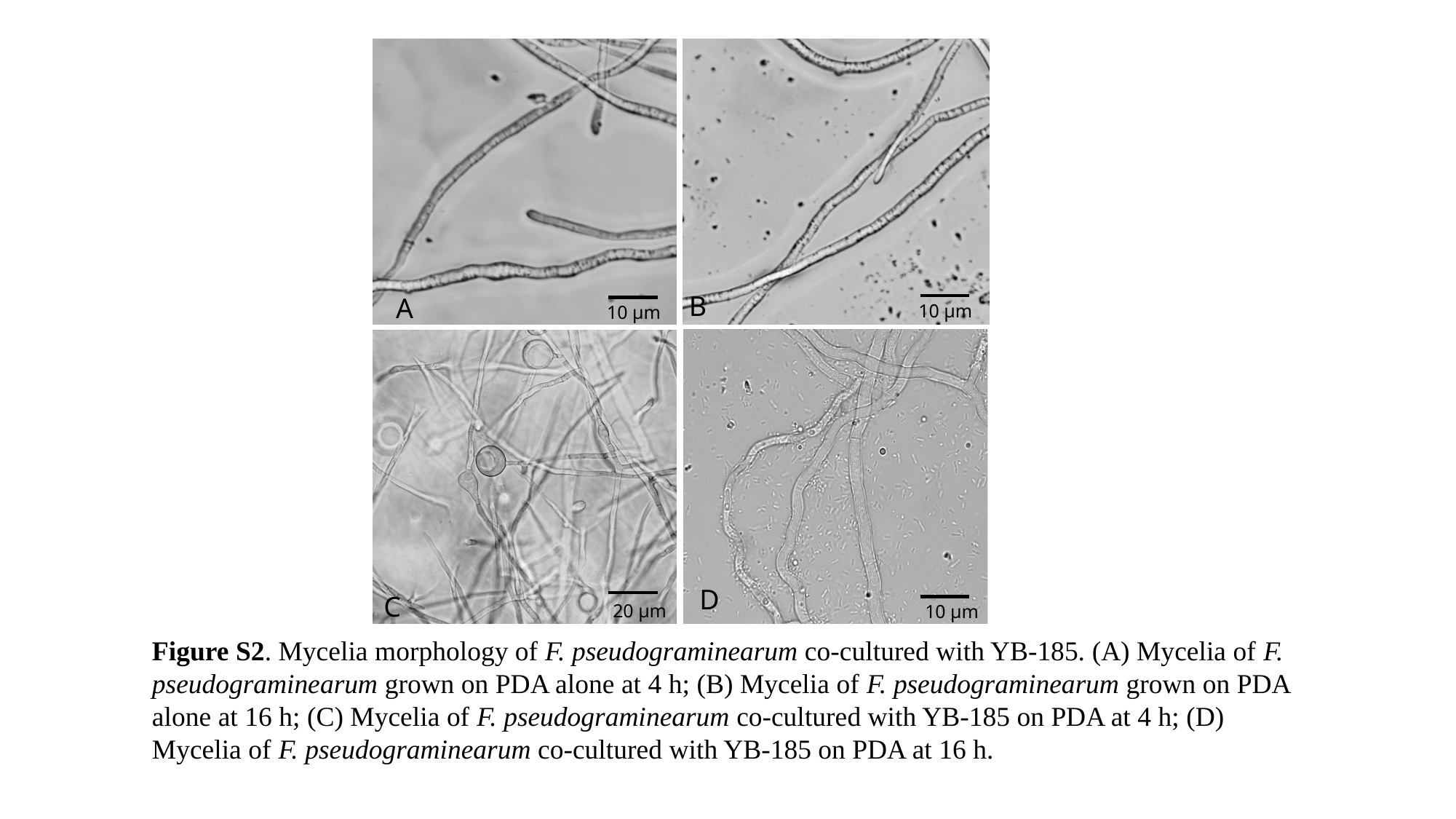

B
A
10 μm
10 μm
D
C
20 μm
10 μm
Figure S2. Mycelia morphology of F. pseudograminearum co-cultured with YB-185. (A) Mycelia of F. pseudograminearum grown on PDA alone at 4 h; (B) Mycelia of F. pseudograminearum grown on PDA alone at 16 h; (C) Mycelia of F. pseudograminearum co-cultured with YB-185 on PDA at 4 h; (D) Mycelia of F. pseudograminearum co-cultured with YB-185 on PDA at 16 h.

## Slide 3
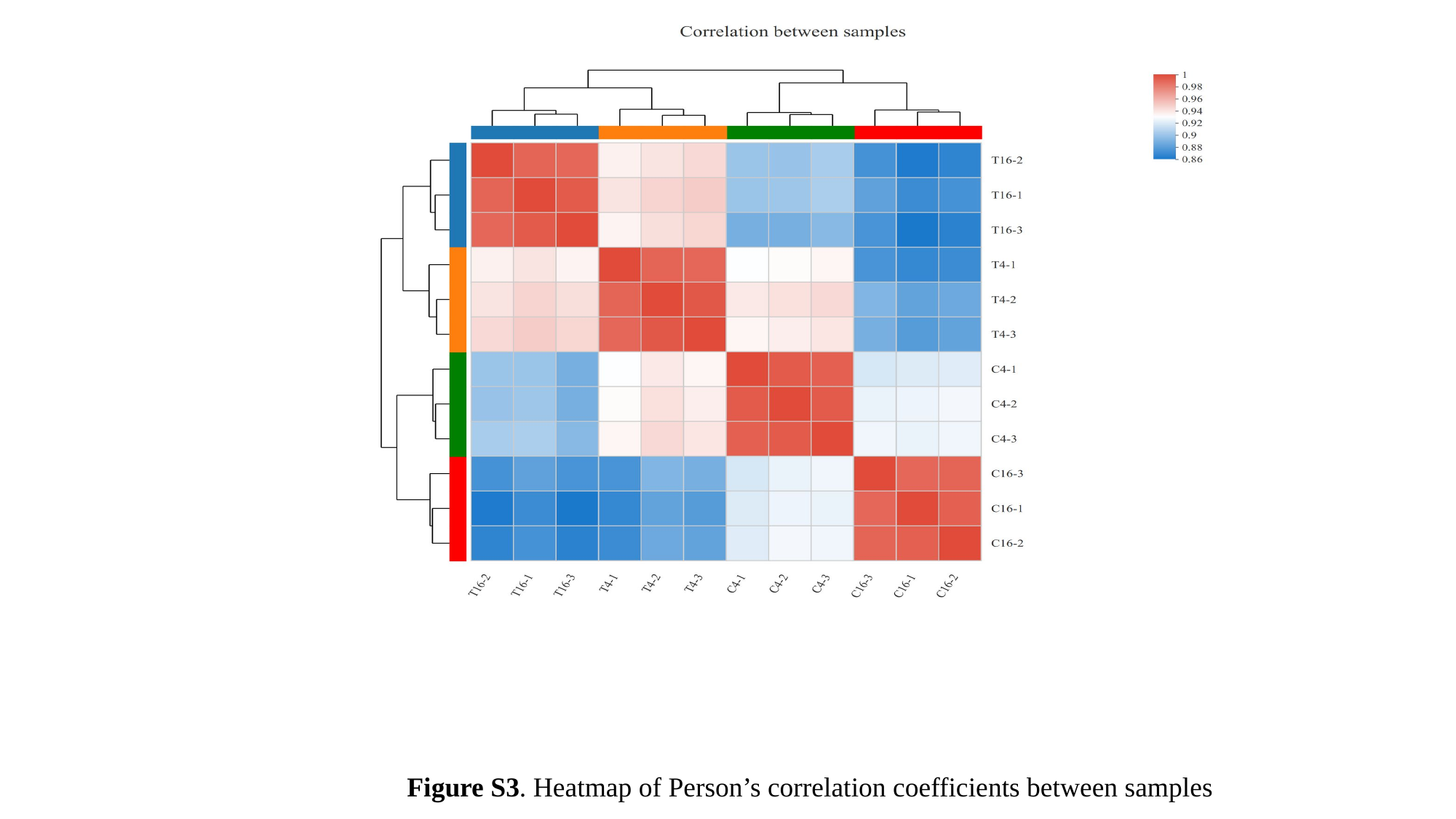

Figure S3. Heatmap of Person’s correlation coefficients between samples

## Slide 4
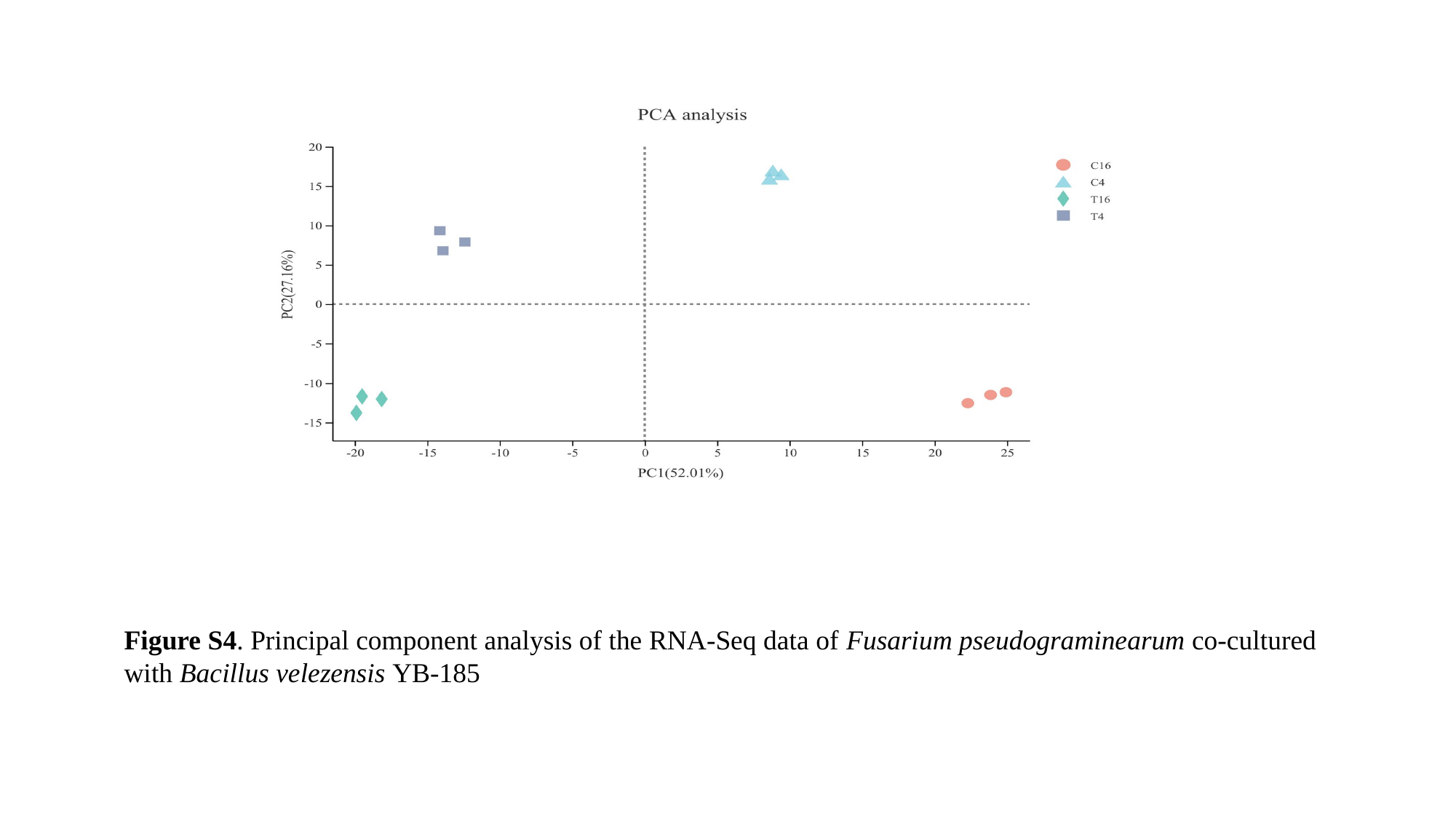

Figure S4. Principal component analysis of the RNA-Seq data of Fusarium pseudograminearum co-cultured with Bacillus velezensis YB-185

## Slide 5
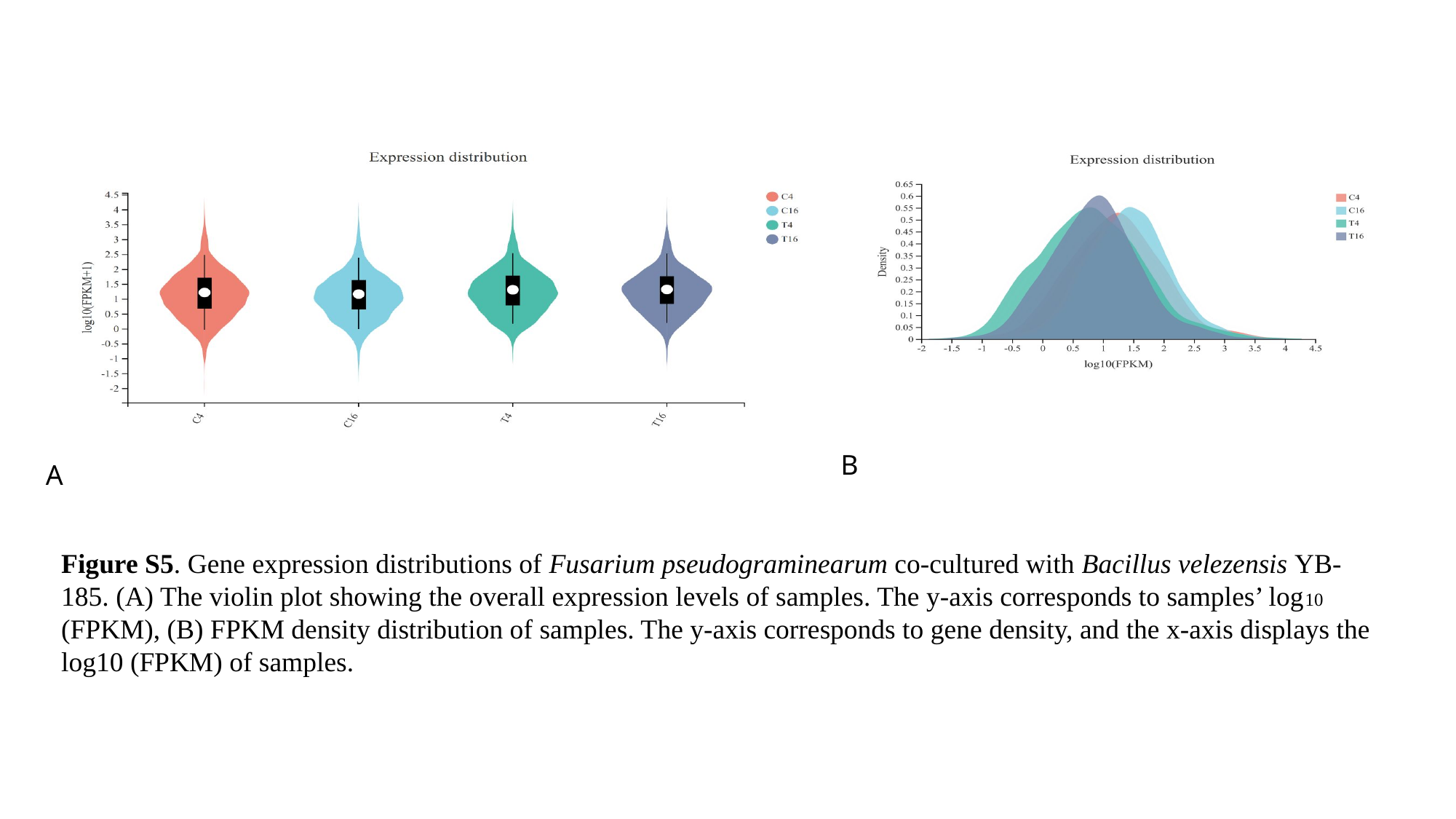

B
A
Figure S5. Gene expression distributions of Fusarium pseudograminearum co-cultured with Bacillus velezensis YB-185. (A) The violin plot showing the overall expression levels of samples. The y-axis corresponds to samples’ log10 (FPKM), (B) FPKM density distribution of samples. The y-axis corresponds to gene density, and the x-axis displays the log10 (FPKM) of samples.

## Slide 6
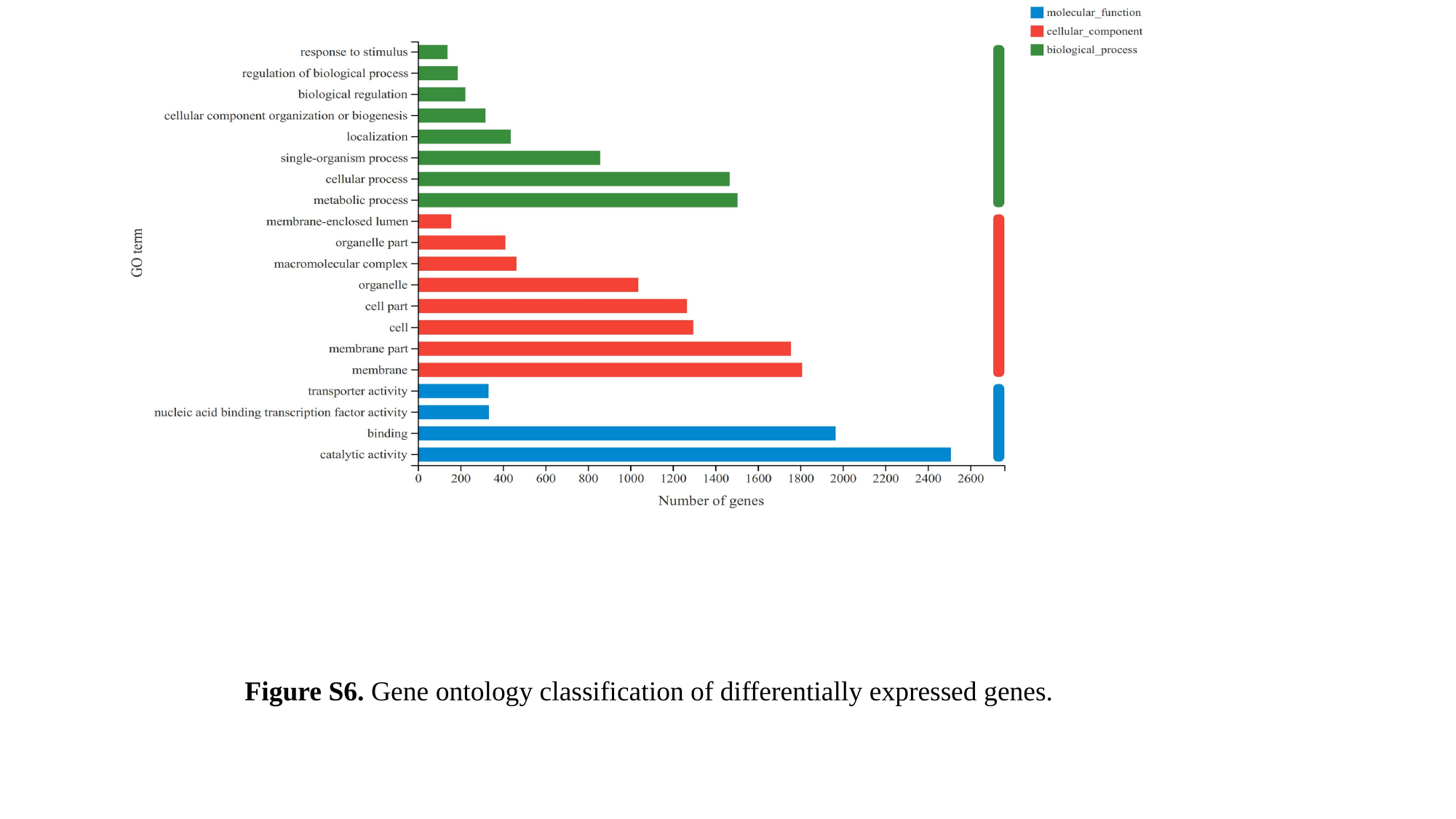

Figure S6. Gene ontology classification of differentially expressed genes.

## Slide 7
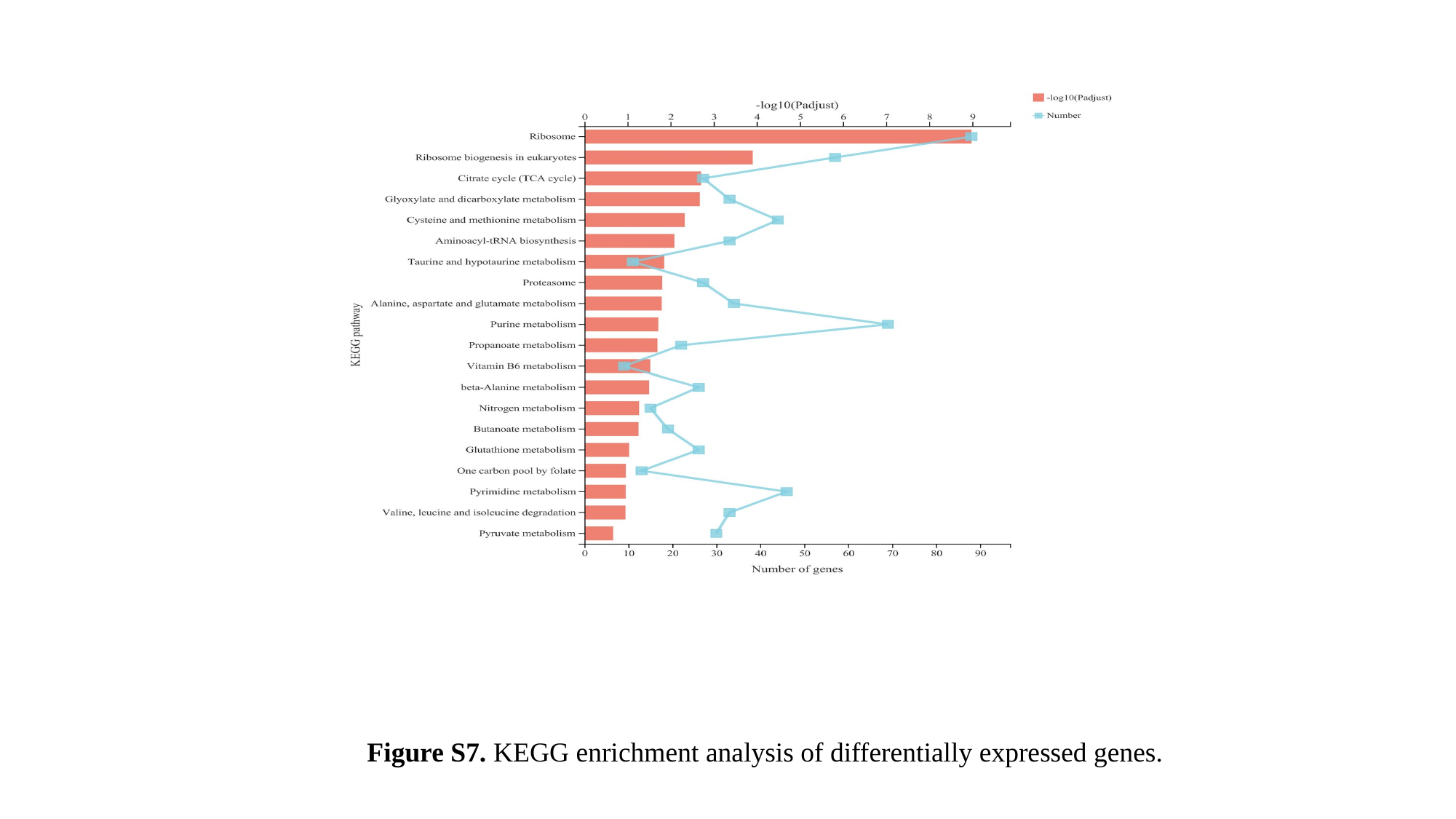

Figure S7. KEGG enrichment analysis of differentially expressed genes.

## Slide 8
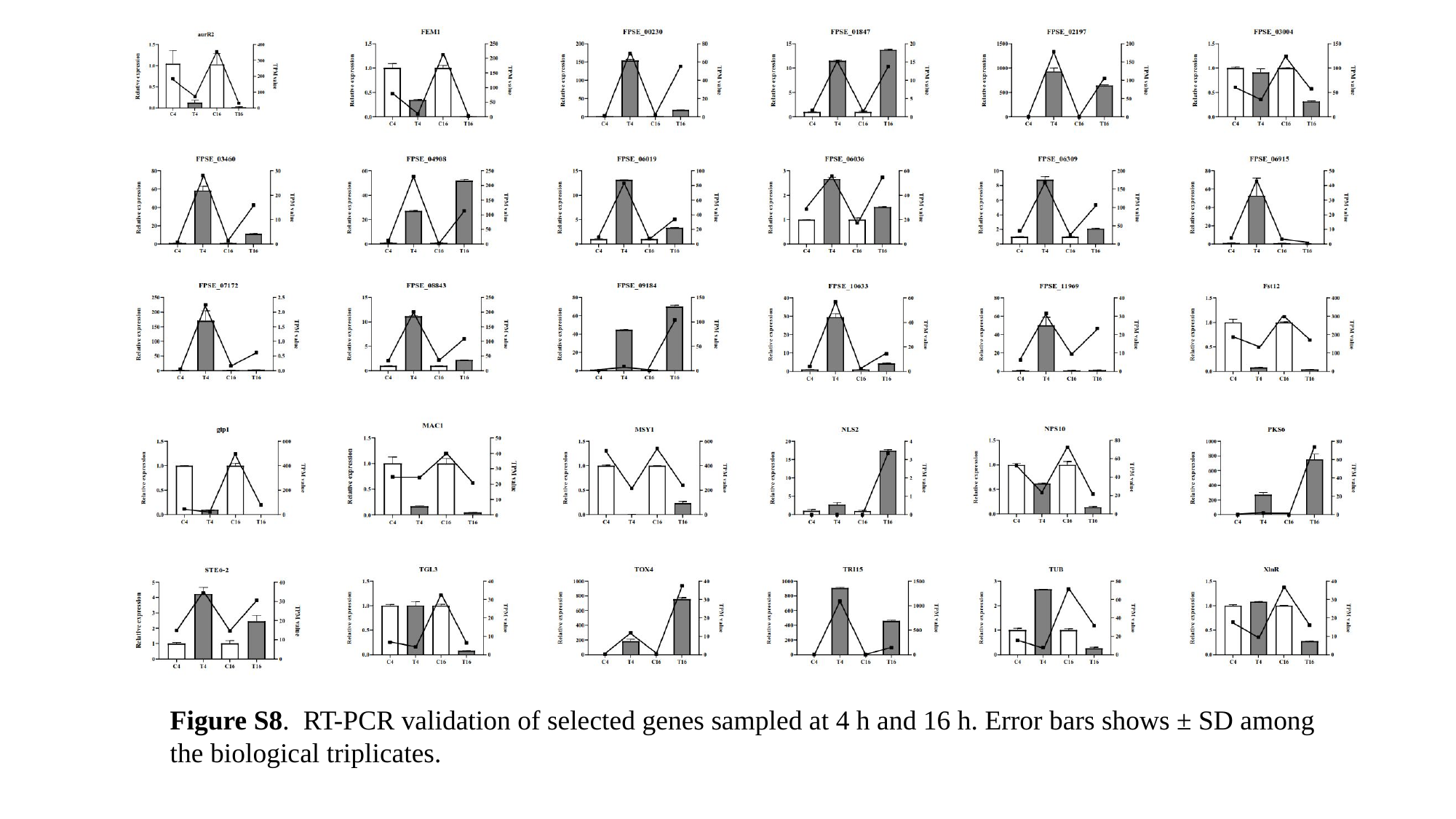

Figure S8. RT-PCR validation of selected genes sampled at 4 h and 16 h. Error bars shows ± SD among the biological triplicates.
